# Lipidation Alters the Structure and Hydration of Myristoylated Intrinsically Disordered Proteins

**DOI:** 10.1021/acs.biomac.2c01309

**Published:** 2023-02-09

**Authors:** Jingjing Ji, Md Shahadat Hossain, Emily N. Krueger, Zhe Zhang, Shivangi Nangia, Britnie Carpentier, Mae Martel, Shikha Nangia, Davoud Mozhdehi

**Affiliations:** † Department of Biomedical and Chemical Engineering, Syracuse University, Syracuse, New York 13244, United States; ‡ Department of Chemistry, Syracuse University, Syracuse, New York 13244, United States; § Department of Chemistry, University of Hartford, West Hartford, Connecticut 06117, United States; ∥ BioInspired Syracuse: Institute for Material and Living Systems, Syracuse University, Syracuse, New York 13244, United States; ⊥ Department of Biology, Syracuse University, Syracuse, New York 13244, United States

## Abstract

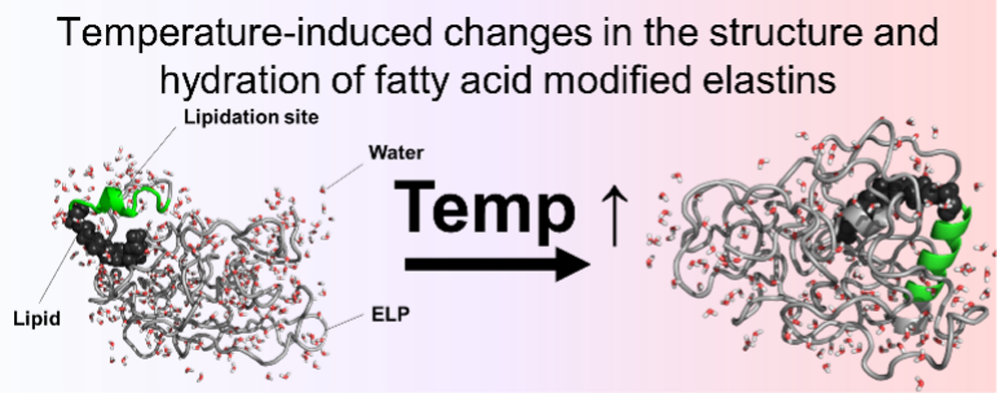

Lipidated proteins are an emerging class of hybrid biomaterials that can integrate the functional capabilities of proteins into precisely engineered nano-biomaterials with potential applications in biotechnology, nanoscience, and biomedical engineering. For instance, fatty-acid-modified elastin-like polypeptides (FAMEs) combine the hierarchical assembly of lipids with the thermoresponsive character of elastin-like polypeptides (ELPs) to form nanocarriers with emergent temperature-dependent structural (shape or size) characteristics. Here, we report the biophysical underpinnings of thermoresponsive behavior of FAMEs using computational nanoscopy, spectroscopy, scattering, and microscopy. This integrated approach revealed that temperature and molecular syntax alter the structure, contact, and hydration of lipid, lipidation site, and protein, aligning with the changes in the nanomorphology of FAMEs. These findings enable a better understanding of the biophysical consequence of lipidation in biology and the rational design of the biomaterials and therapeutics that rival the exquisite hierarchy and capabilities of biological systems.

## Introduction

The development of new peptides/proteins has traditionally focused on engineering their amino acid sequence to regulate the structure–function for desired applications, including materials,^1−9^ vaccines and biopharmaceuticals,^10−14^ sensors,^15,16^ and others.^17−20^ In contrast, nature leverages posttranslational modifications (PTMs)—the decoration of proteins with motifs such as phosphate, carbohydrates, and lipids, among others—to modulate protein structure, function, and location with exquisite spatiotemporal control.^21^ The chemical diversity of PTMs far surpasses the canonical design space of the 20–22 naturally occurring amino acids, exponentially increasing the diversity of proteinaceous molecules available to regulate the spatiotemporal flow of life-sustaining matter, energy, and information.

Utilization of PTMs offers a promising direction for the construction of hybrid protein materials with diversified physicochemical properties, expanded engineering capabilities, and/or altered biological behavior.^22−24^ For instance, lipidation of stimuli-responsive intrinsically disordered proteins (IDPs) has been used to combine the hierarchical assembly of lipids with the temperature-responsiveness of IDPs to create assemblies whose nano- and mesoscale structure can change with temperature.^25−28^ Specifically, fatty-acid-modified elastin-like polypeptides (FAMEs) can form a diverse palette of spherical and anisotropic structures as a function of temperature including spherical nanoparticles^26,29,30^ that can change size^31^ or transition into nanoworms^32^ or fibers.^33^ The ability to reprogram the nanoassembly of these hybrid biomaterials as a function of temperature is desirable for biomedical applications, including drug delivery.^34,35^ Because temperature can be easily and precisely modulated as a therapeutic modality, this thermoresponsiveness can be harnessed to regulate the transport and localization of carriers and encapsulated drugs while causing minimal damage to healthy tissues.^36^

Fulfilling the therapeutic potential of these hybrid nano-biomaterials, however, requires advances in our understanding of the underlying mechanisms of their stimuli-responsive behaviors.^37^ Intriguingly, despite the similarities between unmodified elastins and synthetic coiled polymers, the assembly of FAMEs is inconsistent with the assembly of synthetic polymeric amphiphiles such as lipidated poly(*N*-isopropylacrylamides).^38−40^ In particular, lipidated proteins such as FAMEs are highly asymmetric—14 carbon tail vs 10–30 kDa head group—and therefore morphological changes in the nanostructure of these assemblies are inconsistent with the results of theoretical and experimental studies on polymeric surfactants having small hydrophobic weight fractions, which only form spherical micelles. This inconsistency raises a fundamental question why do some FAMEs nanoassemblies change their size and shape?

**Figure 1:**
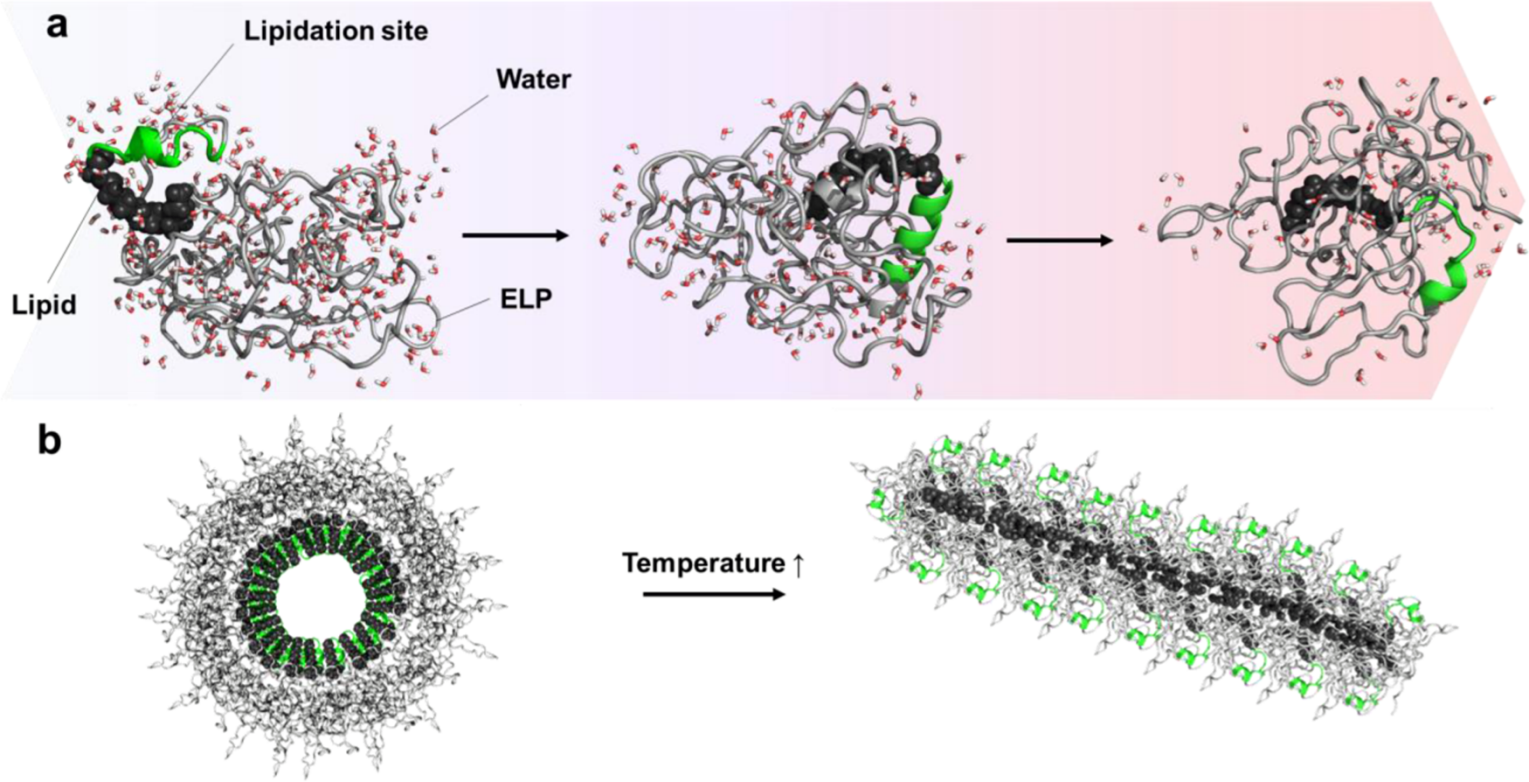
Schematic of temperature-triggered change in the FAME’s structure and nanoscale organization. (a) Temperature and molecular syntax of FAMEs alter the solvent accessibility of the lipid, the secondary structure of the lipidation site, and the hydration pattern of the protein. (b) Schematic of temperature-induced changes in the quaternary organization of FAMEs assembly.

We hypothesized that lipidation alters the energetic and structural interplay between and among FAME’s three domains—lipid, lipidation site (LS), and protein—as a function of temperature, thus modulating the interactions of these domains with each other and with solvent (i.e., hydration), Figure 1.

To test our hypotheses, we used molecular simulations and experiments to study a library of FAMEs in which the length of the disordered protein was systematically varied between 20 and 60 ELP repeat units. We used biophysical and soft-matter characterization techniques to evaluate the FAMEs structure, hydration, and quaternary organization as a function of temperature to build a detailed molecular picture of how lipidation influences the emergent temperature-dependent assembly of this class of hybrid materials. Understanding these molecular details would reveal not only how lipidation alters the biophysical properties of proteins but also new possibilities to make genetically encodable amphiphiles with tailored shapes and functional capabilities such as targeting, drug release, and internalization.

## Materials and Methods

### Materials

The DNA oligonucleotides were synthesized by Integrated DNA Technologies (Coralville, Iowa). The pETDuet-1 vector was purchased from EMD Millipore (Billerica, MA). The chemically competent *Escherichia coli* strains (NEB 5-α, T7 Express *lysY*, and BL21(DE3)), restriction enzymes, ligase, and corresponding buffers, and DNA manipulation and purification kits (Q5-site directed mutagenesis, Monarch gel extraction) were all purchased from New England Biolabs (Ipswich, MA). Isopropyl β-d-1-thiogalactopyranoside (IPTG) was purchased from GoldBio (St. Louis, MO). Apomyoglobin, adrenocorticotropic hormone (ACTH), sinapinic acid, and α-cyano-4-hydroxycinnamic acid (CHCA) were purchased from Sigma-Aldrich (St. Louis, MO). Trifluoroacetic acid (TFA) was purchased from VWR International LLC (Radnor, PA). Yeast extract, tryptone, sodium chloride, high-performance liquid chromatography (HPLC)-grade acetonitrile, dimethyl sulfoxide (DMSO), butanol, isopropanol (IPA), ethanol, myristic acid, SnakeSkin dialysis tubing with 3.5k nominal molecular weight cutoff (MWCO), mass spectroscopy-grade Pierce trypsin protease, ampicillin, kanamycin, and phosphate-buffered saline (PBS) were purchased from Thermo Fisher Scientific (Rockford, IL). 4× Laemmli sample buffer, Mini-PROTEAN TGX Stain-Free Precast Gels, Precision Plus Protein All Blue Pre-stained Protein Standard, and Precision Plus Protein Unstained Protein Standards were purchased from Bio-Rad Laboratories, Inc. (Hercules, CA). The Quantifoil copper grids (Q3100CR1.3) for Cryo-transmission electron microscopy (cryo-TEM) were purchased from Electron Microscopy Sciences (Hatfield, PA). Deionized water was obtained from a Milli-Q system (Millipore SAS, France). Simply Blue SafeStain was purchased from Novex (Van Allen Way Carlsbad, CA). All chemicals were used as received without further purification.

### Molecular Dynamics

The atomistic structures of disordered peptides (GVGVP)*_n_* (*n* = 20, 30, 40, 50, and 60, respectively), the recognition sequence (GLYASKLFSNLG), and eight histidine residues were obtained from Iterative Threading ASSEmbly Refinement (I-TASSER) server.^41−43^ Systems were subjected to a series of energy minimization and equilibration steps with input files generated from CHARMM-GUI solution builder,^44−46^ where disordered peptides were allowed to be modified by myristic acids to generate lipidated peptide systems: myr-V_20_, myr-V_30_, myr-V_40_, myr-V_50_, and myr-V_60_. The CHARMM36m force field^47^ parameters were used for lipidated proteins, salt (0.15 M NaCl), and explicit TIP3P water. The atomistic molecular dynamics (MD) simulations of myr-V_20_, myr-V_30_, and myr-V_40_ were carried out using Anton 2.^48^ The atomistic MD simulations of myr-V_50_ and myr-V_60_ were carried out using GROMACS 2019.^49^ Each system was energy minimized, followed by equilibration in isothermal-isochoric (NVT) and isothermal-isobaric (NPT) for 1 ns each, and production MD run under NPT conditions for 4 μs. Heavy atoms of lipidated proteins were restrained during NVT and NPT equilibration. All restraints were removed during the production MD. The temperature was maintained at 250 K using the v-rescale thermostat^50^ with τ_t_ = 1.0 ps. In the pre-production NPT run, isotropic pressure of 1 bar was maintained using Berendsen barostat^51^ with τ_p_ = 5.0 ps and compressibility of 4.5 × 10^–5^ bar^–1^. In the production MD, we used the Parrinello–Rahman barostat^52^ with τ_p_ = 5.0 ps and compressibility of 4.5 × 10^–5^ bar^–1^. A 2 fs time step was used, and the nonbonded interaction neighbor list was updated every 20 steps. A 1.2 nm cutoff was used for electrostatic and van der Waals interactions. The long-range electrostatic interactions were calculated using the particle-mesh Ewald (PME) method after a 1.2 nm cutoff.^53^ Three-dimensional (3D) periodic boundary conditions (PBC) were applied to each system. The bonds involving hydrogen atoms were constrained using the linear constraint solver (LINCS) algorithm. A similar procedure was used to simulate constructs at 260, 275, 295, 310, and 335 K, respectively. Other than temperature, simulation parameters remained unchanged. Molecular visualization and images were rendered using PyMol^54^ and VMD^55^ software suites. Data analysis and plotting were performed using in-house python scripts based on publicly hosted python packages, such as matplotlib, scipy, and MDAnalysis.^56^ Variables in Figures 4 and S11 are defined as follows: (1) *N*_w_ is the number of H_2_O molecules within the 3.15 Å of peptide chain; (2) *N*_pp_ is the number of intramolecular contacts when the heavy atoms of residues are closer than 6 Å.

### Hydration Analysis

We evaluated the amphiphilicity of the equilibrated structures using annealing simulations of water from the surface of the lipidated protein. The simulation box for the annealing runs consisted of the 4 μs equilibrated lipidated protein and water molecules in the protein’s first hydration shell. The backbone and side-chain heavy atoms of the lipidated proteins were restrained using a force constant of 1000 kJ/mol/nm^2^, while the water molecules were unrestrained. The volume of the simulation box was kept constant, and the system was heated from the initial temperature *T*_dw,i_ = 250 K to a temperature of *T*_dw,f_ = 400 K at the rate of 1 K/20 ps over a 3 ns simulation.

### Cloning

Q5-site-directed mutagenesis kit was used to remove the N-terminal 6xHis tag in the coding sequence of two vectors before the construction of FAME plasmids: (1) an in-house pETDuet-1 vector harboring the gene for *Saccharomyces cerevisiae* NMT (Δ1-35, accession # P14743);^29^ (2) a pET24-a plasmid harboring the gene for (GVGVP)_30_ (Addgene plasmid # 67014).^57^ For each construct, NEBaseChanger was used to design the necessary oligonucleotides (summarized in Table S1). A gradient polymerase chain reaction (PCR) experiment was conducted to empirically identify the optimal annealing temperature (53–70 °C), using the software recommendation as the starting point. After 25 cycles, amplicons were examined by agarose gel electrophoresis, 1% w/v agarose, and Tris–acetate–ethylenediaminetetraacetic acid (EDTA) buffer (130 V, 30 min) and visualized using SYBR SAFE DNA dye. The product corresponding to the linearized vector was then excised from the gel and isolated using a gel extraction kit. The linear product was then incubated with a three-enzyme mix (kinase, ligase, DnpI (KLD)) to circularize the linear amplicon before transformation into Eb5-α cells. After selection over antibiotic plates (ampicillin was used for pETDuet-1 and kanamycin was used for pET24a), sanger sequencing was used to screen for mutants lacking the N-terminal 6xHis tag. The modified pETDuet-1 vector was used as the parent plasmid to construct all FAME plasmids (summarized in Table S2).

A modular cloning strategy was used to create a series of bicistronic vectors (derived from pETDuet-1) to co-express yeast NMT and ELPs fused to the chimeric lipidation site in *E. coli* (Table S2). In these vectors, the multiple cloning site 1 contains a codon-optimized yeast NMT, while the ELP fused to the lipidation site (GLYASKLFSNLGHHHHHHHH) is placed in the multiple cloning site 2. The gene for (GVGVP)_30_ was obtained using PCR from Addgene (plasmid # 67014) and the plasmid containing the gene for (GVGVP)_20_ was a generous gift from the Chilkoti group. These two plasmids were used to synthesize ELPs with 40, 50, and 60 repeats using two rounds of recursive directional ligation.^58^ All cloning steps were conducted in NEB 5-α strains, and sanger sequencing and restriction digest mapping were used to confirm the identity of plasmids.

### Protein Expression

myr-V_30_, myr-V_40_, myr-V_50_, and myr-V_60_ were expressed in *E. coli* BL21(DE3) strains. After growing the cells in a 50 mL of starter 2× YT media (supplemented with 100 μg/mL of ampicillin) at 37 °C for 16–18 h, 4 mL of starter was used to inoculate each L of 2× YT culture media (supplemented with 100 μg/mL of Ampicillin). Cells were grown at 37 °C to OD_600_ of 0.8–1 before adding myristic acid (200 μM). 15 min later, protein expression was induced by adding IPTG (1 mM). After addition of IPTG, the temperature was reduced to either 18 °C (for myr-V_30_) or 28 °C (for myr-V_40_, myr-V_50_, myr-V_60_). Cells were harvested 16 h post-induction by centrifugation (3745*g*, 20 min, 4 °C). The harvested pellet was resuspended in isopropanol (IPA), 4 mL/g of pellet, using a vortex mixer. After clarification of suspension by centrifugation (23 000*g*, 10 min, 4 °C), the supernatant was transferred to a new centrifuge tube. FAMEs were then precipitated by addition of acetonitrile (70% v/v), and collected by centrifugation (23 000*g*, 10 min, 4 °C). The supernatant was discarded, and the protein pellet was solubilized in 50% (v/v) ethanol. Proteins were further purified by reversed-phase HPLC (RP-HPLC) to ensure >95% purity. Acetonitrile was removed by dialyzing the protein solution against water using SnakeSkin Dialysis Tubing (MWCO 3.5 or 7 kDa) for ∼18 h. The proteins were then lyophilized and stored at −20 °C.

myr-V_20_ was expressed in T7 Express lysY Competent *E. coli* cells to eliminate the unwanted leaky expression of protein and the degradation of its N-termini lipidation site prior to induction. The expression and purification protocols were modified as described below: Myristic acid and IPTG were added at a lower cell density (OD_600_ of 0.5–0.8). After induction, the cells were incubated at 28 °C for 7 h. A mixture of IPA/butanol (50% v/v) was used for cell lysis. After precipitation of the protein with acetonitrile, the pellet was resuspended in a mixture of ethanol: acetonitrile: 0.1% TFA in water (2:1:2 (v/v)) and purified by RP-HPLC.

### RP-HPLC

RP-HPLC was performed on a Shimadzu instrument equipped with a photodiode array detector on C18 columns (Phenomenex Jupiter 5 μm C18 300 Å, 250 × 4.6 mm^2^ and Phenomenex Jupiter 5 μm C18 300 Å, 250 × 10 mm^2^). The mobile phase was a linear gradient of acetonitrile and water (0–90% acetonitrile over 40 min, each phase supplemented with 0.1% TFA). The flow rate was 1 mL/min for analytical HPLC and 4.7 mL/min for preparative HPLC. HPLC traces were background corrected by subtracting the absorbance of a blank trace (resulting from water injection on the same column and eluent gradients) from the sample’s trace. The traces in Figure S4b were obtained using a C4 column (Phenomenex Jupiter 5 μm C4 300 Å, 250 × 4.6 mm^2^) using the same mobile phase gradient.

### Matrix-Assisted Laser Desorption/Ionization Coupled to Time-of-Flight Mass Spectrometry (MALDI-TOF-MS)

MALDI-TOF-MS was conducted on Bruker microflex LRF with a microScout ion source using α-cyano-4-CHCA or sinapinic acid as matrix. Apomyoglobin (*M*_W_ = 16 952 Da) was used for calibration. To confirm the regioselectivity of lipidation, proteins (0.9 nmol) were digested with trypsin (1 μg) for 3 h using 50 mM ammonium bicarbonate (pH = 7.8) as a buffer. The resulting peptide fragments were analyzed by MALDI-TOF-MS using CHCA as the matrix and ACTH as calibrant ([M + H]^+^ = 2465.1989, monoisotopic).

### Dynamic Light Scattering (DLS)

DLS was performed on a Zetasizer Nano (Malvern Instruments, U.K.) with the 173° backscatter detector. All sample preparation steps were conducted at 277 K, and samples were equilibrated at each temperature for 10–15 min before measurement. Protein solutions were prepared in PBS to the final concentration of 20 μM and passed through a poly(vinylidene difluoride) (PVDF) filter (0.45 μm). Samples were then transferred to a DLS cuvette (also precooled at 277 K), and quickly transferred into the Zetasizer. After incubation at 288 K, the first set of DLS measurements was conducted. Subsequent measurements were conducted at alternating temperatures (heating to 298 K and cooling to 288 K) for three cycles. Measurements were performed in triplicate at each temperature. The hydrodynamic diameter (*Z*_avg_) and polydispersity index (PDI) were calculated by cumulant fit of the autocorrelation function. We point out that *Z*_avg_ is an “effective” hydrodynamic radius. For nonspherical particles (such as those formed by FAMEs after thermal treatment), *Z*_avg_ provides an approximation of their size. This shortcoming, however, is compensated by the robustness and reproducibility of *Z*_avg_ (even when applied to nonspherical particles), as the correlation functions are analyzed with minimal information/assumptions.^59^

### Differential Interference Contrast (DIC) Microscopy

Protein coacervates were imaged with DIC using a Zeiss AxioObserver Z1 widefield microscope (Carl Zeiss Inc., Berlin, Germany) connected to an ORCA-Flash4.0 LT+ Digital CMOS camera (Hamamatsu Photonics, Hamamatsu, Japan). After incubation at 308 K for 10 min, the protein solutions (50 μM in PBS) were deposited onto a glass slide which was shielded with a coverslip. Images were processed and analyzed using MetaMorph software (Molecular Devices, version 7.8.8.0) and ImageJ (NIH, version 1.53f51).

### Cryo-Transmission Electron Microscopy (Cryo-TEM)

**Figure 2:**
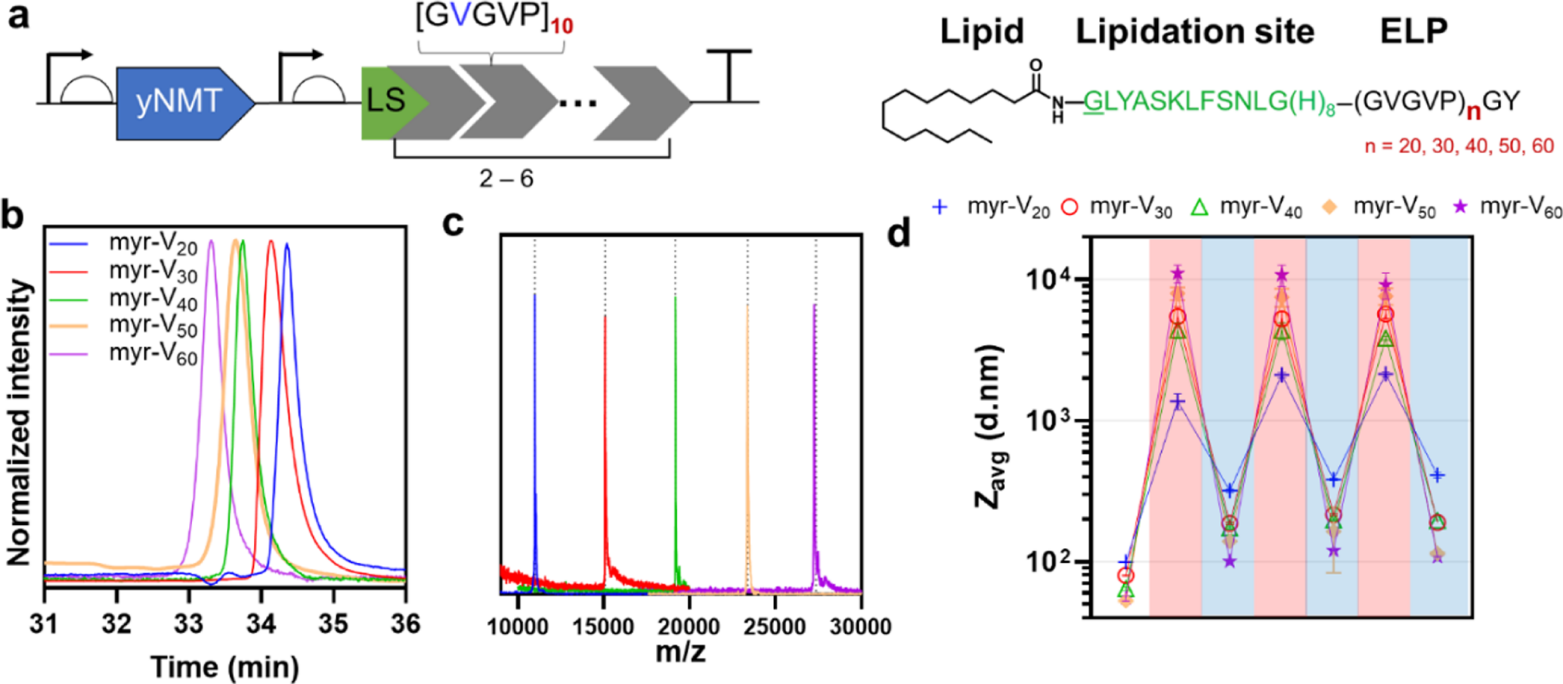
Molecular and soft-matter characterization of FAME library. (a) Architecture of expression units used for the biosynthetic production of FAMEs library. Each FAME contains a myristoylated lipidation site (LS), M-GLYASKLFSNLG, followed by an octahistidine tag (8xHis) and fused to ELPs with different numbers of GVGVP repeat units (i.e., 20, 30, 40, 50, and 60). (b) Analytical reversed-phased HPLC confirms the purity of recombinant FAMEs. The retention time of FAMEs is inversely proportional to the ELP length, suggesting a decrease in the overall hydrophobicity as the ELP length increases. (c) Mass spectrometry confirms the identity of each protein and the regioselective addition of a single myristoyl group to the N-terminal residue (Figure S2). (d) Dynamic light scattering is used to monitor the assembly of FAMEs as a function of temperature. FAMEs are dissolved in cold phosphate-buffered saline (PBS) (288 K, unshaded area) and are subsequently heated and cooled to 298 K (above *T*_t_, shaded in red) and 288 K (below *T*_t_, shaded in blue), respectively. The average hydrodynamic diameter of FAMEs increases after the first cycle of heating/cooling but remains unchanged in subsequent cycles. Error bars are standard deviations of three measurements.

Protein solutions were prepared in PBS (20 μM) at 277 K. The solutions were then incubated under two conditions: (1) 288 K for 10 min (corresponding to Figure 6a–d) and (2) 308 K for 10 min, followed by incubation at 288 K for 15 min (corresponding to Figure 6i–l). After thermal processing, samples were applied to TEM grids, which were previously plasma-treated (Pelco easiGlow, negative polarity, 45 s, 30 mA) to render them hydrophilic. After plunge freezing in liquid ethane, grids were imaged on a Tecnai BioTwin 120 kV transmission electron microscope. Images were collected on a Gatan SC1000A charge-coupled device (CCD) camera and analyzed using ImageJ (NIH, version 1.53f51). To improve the resolution of corona, the cryo-TEM images of fibers have been processed with ImageJ as described below. The images are first denoised using despeckle plugin (median filter) followed by fast Fourier transform (FFT) band-pass filter (filter up to 10 pixels, down to 400, no stripe suppression, with autoscale and saturation). Finally, the background was subtracted through the rolling ball radius method (400 pixels). The raw and processed TEM images are provided in the Supporting Information (Figures S17–S22).

## Results and Discussion

### Design, Biosynthesis, and Characterization of FAME Library

To address our fundamental question, we designed and biosynthesized a library of FAMEs with systematically varied length, fused to an identical lipid tail (myristoyl, C14:0) and lipidation site. This was achieved by co-expressing a yeast lipidation enzyme (*N*-myristyoltransferase, NMT) with ELPs fused to the peptide substrate of this enzyme (i.e., lipidation site), Figure 2a.^60^

NMT specifically prefers myristoyl-CoA as the lipid donor, and except for the presence of N-terminus Gly in the substrate proteins, the sequence requirements for myristoylation remain debated. Bioinformatics analysis suggests sequence biases in the first 17 residues of myristoylated proteins, i.e., some amino acids are (dis)favored in specific positions.^61,62^ Work by Gordon and co-workers using short peptides suggested that the first eight amino acids may contain the necessary information for recognition and lipidation by NMT.^63^ The crystal structure of NMT further reinforced this idea by showing that binding of activated lipid to NMT induces formation of a tunnel-like cavity for the peptide substrate.^64^ The first eight residues of a substrate fit into this binding pocket and form intimate contacts with the enzyme. The more subtle restrictions in the other positions are due to the formation of secondary interactions/contact with the tip of the tunnel or with the surface-bound residues of NMT.

Based on this information, we created a chimeric recognition sequence that contains the first 11 residues from yeast ADP-ribosylation factor 2 (ARF2), a naturally myristoylated regulatory GTPase, followed by an octahistidine tag (8xHis), resulting in the N-terminal sequence of GLYASKLFSNLGH_8_.

Intrinsically disordered protein polymers, such as elastin-like polypeptides used here, are excellent model systems because their repetitive sequence facilitates the parameterization of the protein component into two essential features: the composition of the repeat and its length. The design of the bicistronic vector allows us to swap the ELP gene to generate a library of FAMEs with 20, 30, 40, 50, or 60 repeats of GVGVP pentad fused to an identical lipidation site (LS), Figure S1. Recombinant expression and posttranslational modification yielded the myristoylated FAMEs, hence referred to as myr-V_20_, myr-V_30_, myr-V_40_, myr-V_50_, and myr-V_60_. Although the His tag allows isolation of FAMEs using a metal affinity column, we instead utilized a chromatography-free technique developed by Thompson and co-workers,^65^ which we previously demonstrated successfully purifies post-translationally lipidated elastins.^66^ This method uses isopropanol to lyse cells and isolates ELPs by the addition of the nonsolvent, acetonitrile. For this study, the ELP pellet was redissolved and subjected to reverse-phase high-performance liquid chromatography (RP-HPLC) to achieve >95% purity. The organic solvents used in HPLC also disassembled FAMEs, thus eliminating the (potential) effect of purification process on the assembly of FAMEs.

Analytical RP-HPLC (Figure 2b) confirmed the successful purification of all FAMEs from unmodified ELPs, retention time (*t*_R_) <30 min. Although each lipidation site contains a nucleophilic lysine residue, the HPLC trace did not show any evidence of dual lipidation, i.e., no secondary peak was observed after the main construct. MALDI-TOF-MS was used to confirm the addition of a single myristoyl group to each FAME. the experimentally observed mass-to-charge ratio was in close agreement with the theoretical molecular weight of single lipidated constructs, and no peaks corresponding to unmodified or dual lipidated proteins were observed (Figures 2c and S2 and Table S3). To confirm the regioselectivity of the modification, each FAME was digested with trypsin and the resulting peptide fragments were analyzed using MALDI. In all cases, we observed a peak corresponding to the myristoylated N-terminal peptide fragment, and no unmodified peptide fragment was observed (Figure S3). We are cognizant that MALDI-TOF is not quantitative, but in combination with RP-HPLC, it provides strong evidence that all of the FAMEs constructs are modified with a single myristoyl group, appended to the N-terminal Gly.

The RP-HPLC trace also provides a bulk measure of hydrophobicity for FAME constructs. Contrary to our expectation, the retention time of FAMEs on the C18 column was inversely correlated with their length, *t*_R_ ± 0.1 (min) = 34.4 (myr-V_20_); 34.1 (myr-V_30_), 33.7 (myr-V_40_), 33.6 (myr-V_50_), and 33.3 (myr-V_60_). One interpretation of these results is that the mean hydrophobicity of FAMEs is inversely proportional to the length of the ELP domain, as all FAMEs contain the same hydrophobic lipid. Nonetheless, as shown in Figure S4, the retention times in RP-HPLC are determined by a balance of complex interactions between the analyte, eluent, and immobilized phase.

### Temperature-Dependent Assembly of FAMEs Deviates from the Behavior of Canonical ELPs

Following molecular characterization, we used DLS to investigate the thermoresponsiveness of FAMEs; we monitored changes in the average hydrodynamic diameter of FAMEs as a function of solution temperature (Figure 2d). Unlike ELP block copolymers that only self-assemble above the transition temperature (*T*_t_) of the hydrophobic block,^67−69^ the addition of a single fatty acid was sufficient to drive the self-assembly of all FAMEs even at the lowest experimentally tested temperature (288 K) used, as indicated by the observed hydrodynamic size (50–100 nm) remaining much larger than the theoretical size of unmodified ELPs (∼expected to range from 4 to 8 nm for V_20_ to V_60_). The increase in ELP length (number of pentad-units) reduced both the size and polydispersity index of FAMEs (Figure S5). *Z*_avg_ for myr-V_20_, myr-V_30_, myr-V_40_, myr-V_50_, and myr-V_60_ were 99 ± 6, 80 ± 1, 64 ± 2, 53 ± 1, and 55 ± 2 nm, respectively. The equilibrium size of these aggregates remained fairly stable at temperatures between 288 and 294 K but abruptly increased by 2–3 orders of magnitude over a narrow temperature range. Surprisingly, we observed that *T*_t_’s of FAMEs remained fairly constant irrespective of the length of the ELP domain, 296 ± 1 K, (Figure S6). This result is inconsistent with the behavior of nonlipidated ELPs having a similar sequence. Specifically, both computational and experimental studies indicate that the transition temperature of unmodified ELPs exhibits power-law dependence with length—i.e., (*T*_t_(GVGVP)*_n_* ∼ (*n*)^−0.65^).^70,71^ Moreover, these results are not in agreement with trends based on the mean hydrophobicity approximation as determined by analytical HPLC.

Lipidation also altered the reversibility of temperature-induced aggregation of modified proteins. The phase separation of canonical ELPs is reversible when the temperature is reduced below their respective *T*_t_. While FAME solutions became clear after cooling samples to 288 K, interestingly DLS showed that the *Z*_avg_ for all FAMEs increased compared to the initial sample, pointing to an irreversible change in the nanostructure of lipidated constructs. We investigated whether these increases in size can be repeated by subjecting the samples to two additional cycles of heating to 298 K and cooling to 288 K and measuring the FAMEs’ sizes at the end of each heating and cooling cycle. As shown in Figure 2d, myr-V_30_, myr-V_40_, myr-V_50_, and myr-V_60_ no longer exhibited an irreversible increase in size following either of the two additional heating/cooling cycles. However, the size of myr-V_20_ continued to increase in the second and third cycles, by 20 and 30%, respectively. This shows that while the phase separation of FAMEs is reversible macroscopically across multiple cycles of heating and cooling, there is an irreversible change in the nanoassembly of structures in the first cycle, and more importantly, the ELP length determines the size and stability of nanostructures as a function of temperature. The thermal treatment amplified the differences between the size of different constructs but did not alter the general length-dependence trends in size (Figure S7): myr-V_20_ formed the largest nanoparticles (411 ± 14 nm); both myr-V_50_ and myr-V_60_ formed the smallest nanoparticles (114 ± 1 and 108 ± 1 nm), while myr-V_30_ and myr-V_40_ were intermediate in size (190 ± 1 and 196 ± 1 nm, respectively). The similarities in the assembly behavior of myr-V_50_ and myr-V_60_ (both before and after thermal treatment) are consistent with the power-law scaling of ELP conformational properties.^68^ Therefore, in the remainder of this paper, we focus our discussion on myr-V_20_, myr-V_30_, myr-V_40_, and myr-V_60_. The data for myr-V_50_, which, as expected, behaves similarly to myr-V_60_, are provided in the corresponding Supporting Figures.

### Computational Nanoscopy Provides a Window into the Molecular Details of FAME Thermoresponse

To understand the molecular origins of temperature-triggered change in FAME assembly, we used molecular dynamics (MD) simulations to evaluate the temperature-induced changes in the structure and hydration pattern of FAMEs. We performed all-atom MD simulations for FAMEs of different ELP lengths in explicit water (with 150 mM of sodium chloride) at 250–335 K for 4 μs. These results align with the experimental protocol: Start at low temperature (*T* < *T*_t_) to identify a FAME’s equilibrium structure, a condition that mirrors the behavior of a FAME when first dissolved in buffers at low temperatures. Then, increase the temperature above *T*_t_ to identify changes in the structure and hydration of FAMEs.

### Interplay between the Secondary Structure of the Lipidation Site and ELP Length

**Figure 3:**
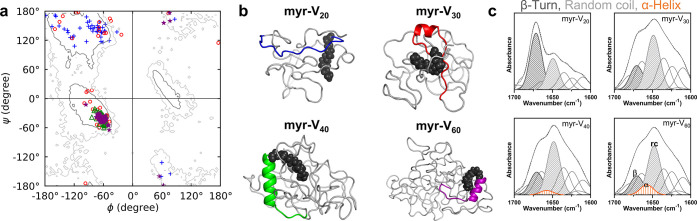
ELP length alters the propensity of the lipidation site to adopt a stable secondary structure. (a) Ramachandran plot (ϕ, ψ dihedral angle distributions) for the backbone residues in the lipidation site in myr-V_20_ (blue plus symbols), myr-V_30_ (red open circles), myr-V_40_ (green open triangles), and myr-V_60_ (purple filled stars), at 295 K over last 1 μs of the MD simulation. In myr-V_20_, the lipidation site remains disordered, evident in the spread of data points across the parallel and antiparallel β sheets regions of the Ramachandran angle space. In myr-V_30_, the lipidation site adopts a partial helical structure, while in myr-V40 and myr-V_60_, the lipidation site adopts a well-defined right-handed α helix that is stable over the last 1 μs of the simulation. (b) Snapshot of representative FAME structures at 295 K obtained from simulation. The secondary structure of each lipidation site matches the torsional angles obtained from the highest-intensity points in Ramachandran plots. (c) Deconvoluted Fourier transform infrared (FT-IR) spectra of the soluble FAMEs (below transition temperature, 100 μM at in D_2_O) reveal differences in FAMEs’ secondary structures. myr-V_20_ exhibits the highest b-turn content. As ELP length is increased, random coil content is increased, as indicated by the increased intensity of the peak at 1645 cm^–1^. Consistent with MD simulations, only myr-V40 and myr-V_60_ exhibit a peak at 1657 cm^–1^, attributed to α helices in a deuterated environment.

The structures obtained after MD simulations were analyzed to calculate the dihedral angles of the peptide backbone for each residue in the lipidation site of FAMEs at 295 K. Figure 3a (and Figure S8) shows the Ramachandran plots constructed for the lipidation site based on these dihedral angle calculations. The high-intensity region in the Ramachandran plot represents the most favorable low-energy torsional angles of the peptide backbone for each residue. Even though the lipidation site is identical for all four FAMEs, changing the ELP length altered the Ramachandran plot and consequently the secondary structure propensity of lipidation sites. In myr-V_20_ (blue plus symbols), the dihedral angles corresponding to lipidation site (GLYASKLFSNLGHHHHHHHH) residues were scattered over various conformational spaces in the Ramachandran plots for all temperatures. These dihedral angles indicate that the lipidation site in myr-V_20_ is conformationally flexible irrespective of the temperature of the simulation. In myr-V_30_ (red circles), however, we found that at 295 K, the lipidation site transiently adopts secondary motifs such as the right-handed α helices and β sheets. Interestingly, the Ramachandran plots of the myr-V_40_ (green triangles) and myr-V_60_ (purple stars) were similar, with the torsion angles of lipidation site residues clustered at the right-handed α helical conformational space of Φ from about −50 to −100° and for Ψ from −30 to −70° at 295 K. In comparison, the lipidation site of myr-V_30_ (red circles) was more ordered than myr-V_20_, but less ordered than myr-V_40_ and myr-V_60_, consistent with the overall trend that increasing the ELP length stabilizes the secondary structure of the lipidation site. In contrast to the behavior of lipidation sites, in Ramachandran plots for all ELP-residues (i.e., VPGVG motifs), the location of high-intensity peaks was similar and so independent of ELP length (Figure S9); this indicates that the ELP domains remained highly disordered throughout the simulation.

Figure 3b shows representative structures of four FAMEs at 295 K, corresponding to the highest-intensity points in Ramachandran plots constructed from dihedral angles for each construct’s lipidation site. In these structures, the lipid is visualized as black spheres, while the lipidation site is visualized as a cartoon with colors corresponding to the construct, and the remainder of the ELP is visualized as a gray ribbon. As discussed previously, the lipidation sites of myr-V_20_ and myr-V_30_ remain disordered, while the lipidation sites of myr-V_40_ and myr-V_60_ adopt a helical conformation. The lipidation site of myr-V_30_ samples’ conformations corresponds to both helical and disordered structures. The ELP domains for all four constructs remain disordered.

To experimentally verify MD predictions, we used Fourier transform infrared (FT-IR) spectroscopy to identify and compare the secondary structure elements of FAMEs. Figure 3c shows the amide I band region of the FT-IR spectra of FAMEs dissolved in D_2_O and its deconvolution. We chose D_2_O as the solvent because the H/D exchange results in differentiation of the peak location for the amide carbonyl of residues in random coil and α helix motifs. In water, the amide bonds in both secondary structures absorb at 1654 cm^–1^; however, in D_2_O, the random coil band peak shifts to 1645 cm^–1^, while the α helix peak shows minimal shifts.^72^ Deconvolution of FT-IR bands shows that FAMEs’ secondary structures are consistent with predictions from MD simulations. As expected in all cases, the random coil content of the polypeptide increases with the ELP length. However, only in myr-V_40_ and myr-V_60_ the deconvoluted FT-IR spectra exhibit a peak centered at 1657 cm^–1^, which is attributed to the formation of α helix.

### Temperature-Induced Structural Changes of FAMEs

We then compared in silico structures of FAMEs at elevated temperatures (e.g., 310 and 335 K > *T*_t_) to reveal the changes to the structure of FAME constructs. ELP domains of all FAMEs remained disordered at higher temperatures, and only minor structural transitions, such as increased β-turns at higher temperatures, were observed. This behavior is consistent with previous studies on unmodified ELPs^73^ and shows that lipidation does not change the structural behavior of ELP—hence, the observed differences between the thermoresponsive assembly of ELP and FAMEs are not due to significant differences in the structure of ELP domain. On the other hand, the secondary structure of the lipidation site was dependent on the temperature (Figure S10). The secondary structural motifs observed for myr-V_30_, myr-V_40_, and myr-V_60_ lipidation sites melted over 310–335 K (i.e., temperature-induced order-to-disordered transition) while the disordered lipidation site of myr-V_20_ remained conformationally flexible at high temperatures.

### Temperature-Induced Changes in the Hydration of FAMEs

**Figure 4:**
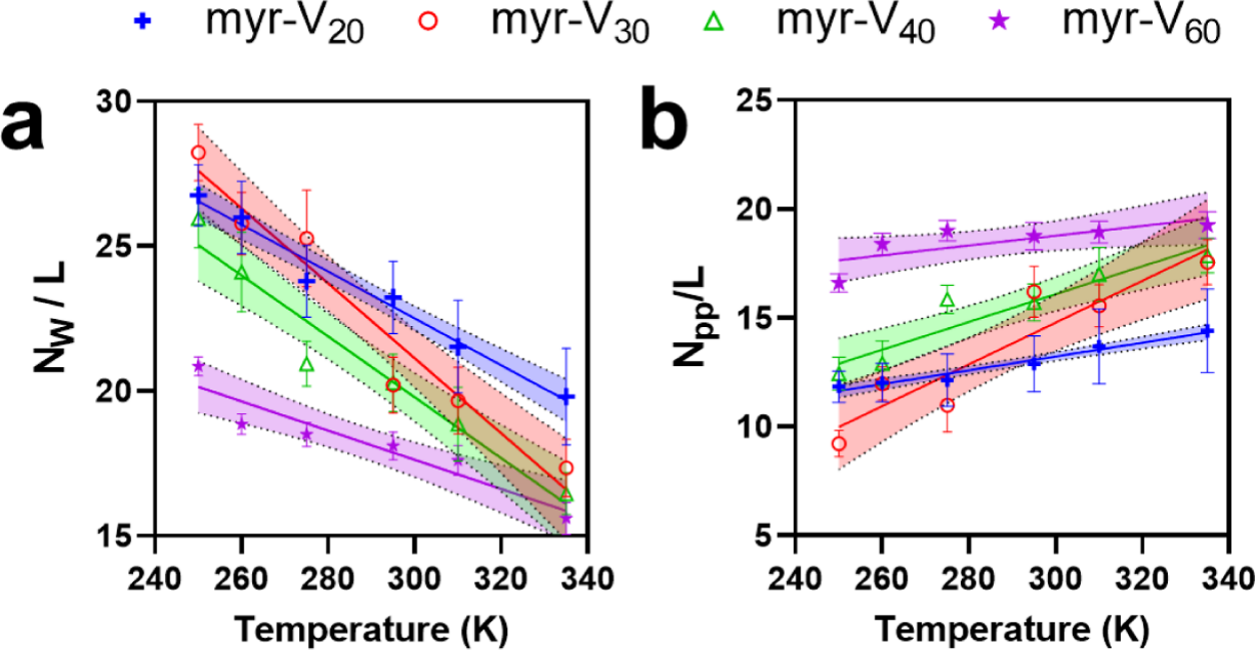
Interplay between lipids and ELP alters the temperature-dependent hydration of FAME chains. Temperature dependence of hydration properties of a single FAME chain. The length-normalized number of water molecules (*N*_w_) within the first hydration shell of peptide backbone versus temperature (a), and the number of intrachain (peptide–peptide) contacts (*N*_pp_) versus temperature (b). Slopes of the linear fit of all of the curves are shown in Table S4. The error bars represent the standard deviation calculated from the time average of each simulation. The dotted lines (and the colored band) represent the 90% confidence interval of the fitted line.

After ruling out the structural changes in the ELP domain as a factor that distinguishes the unmodified and lipidated constructs, we investigated the changes in the hydration of FAMEs as a function of temperature. The lower critical solution temperature behavior of ELPs is intricately linked to the behavior of the hydration shell around the polypeptide, as their thermoresponse is primarily driven by the release of “frozen” water molecules above their *T*_t_.^74−77^ The release of water molecules is concomitant with strengthening interactions between the ELP chain. To quantify these effects, we calculated the number of water molecules in the first hydration shell (*N*_w_) and the number of intramolecular contacts by the polypeptide chain (*N*_pp_) as a function of temperature. Because the absolute value of *N*_w_ and *N*_pp_ depends on the length of the polypeptide (e.g., as the ELP length is increased, the overall number of waters in its hydration shell is increased), Figure 4 (and Figure S11) depicts the length-normalized value of these variables for various constructs as a function of temperature. Two trends are apparent from these results: As temperature increases, (1) the total number of water molecules in the first hydration shell of FAMEs decreases (negative slope), and (2) polypeptide residues form more contacts (positive slope). These trends are consistent with the weakening of protein–water interactions and the strengthening of protein–protein interactions. Despite overall similarities in the behavior of the four constructs, we observed intriguing differences in FAMEs as a function of ELP length. Previous computational studies on unmodified ELPs have shown that temperature-dependent changes in the hydration of ELPs depend only on the physicochemical characteristics of the repeat motif—and not on the length of the ELP.^70^ Put another way, length-normalized changes in *N*_w_ (or *N*_pp_) are expected to be identical to each other as the chemistry of the repeat unit is independent of the length of the polypeptide. However, in contrast to our expectations, the behavior of FAMEs was length-dependent (Figure 4a)—as the curves do not collapse onto each other. In particular, short and long FAMEs (myr-V_20_ and myr-V_60_) exhibited subtle variations compared to mid-length FAMEs (myr-V_30_ and myr-V_40_) behavior. See, for example, the slopes of the linear fits in Figure 4, which indicate the temperature-dependent rate of dehydration for each construct. The more negative slope observed for myr-V_30_ and myr-V_40_ signifies that the mid-length constructs lose more water molecules (dehydrate) as temperature increases (see Table S4 for confidence intervals). We observe similar length-dependent trends when analyzing changes in the number of intramolecular contacts (i.e., after normalization to length) as a function of temperature (Figure 4b). Here, as the temperature is increased, myr-V_30_ and myr-V_40_ form more intramolecular contacts compared to myr-V_20_ and myr-V_60_.

These observations led us to hypothesize that the anisotropic addition of lipidation alters the physicochemical characteristics of pentad repeats along the length of the ELP domain. That is—despite the large asymmetry between the length of ELP and the lipid—the incorporation of this hydrophobic motif at one end of ELP alters the temperature-dependent hydration properties of ELP repeats, rendering pentads with identical compositions essentially different from each other.

### Lipidation Induces “Unique” Hydrophobic Patches in ELP Domains

To investigate this hypothesis, we mapped the hydrophobic patches on each FAME’s structure at various temperatures using MD simulations to develop a functional hydropathy index. Our approach differs from traditional hydropathy scales that rely on a constant value for each amino acid side chain or on the atomic-level hydrophobicity scale that forms the residue, e.g., grand average of hydropathicity index (GRAVY).^78^ Other recent computational studies have highlighted that the amphiphilicity of structured proteins is strongly influenced by their nanoscale chemical and topographical patterns, as these factors alter the behavior of the hydration shell.^79,80^ However, the available methods have not been used for intrinsically disordered protein constructs as in FAMEs. Our hydropathy scale evaluates the hydrophobicity of a residue based on its physicochemical properties and local topography and is suitable for thermoresponsive FAMEs that undergo temperature-induced conformation changes during self-assembly. The scale utilizes a residue’s ability to stay hydrated over increasing temperature to measure its hydropathy. The first step in the hydropathy scale workflow is obtaining an MD-equilibrated FAME structure at a temperature (*T*_MD_). Second, the FAME structure and the water molecules in the protein’s first hydration shell were transferred to the center of a cubic simulation box. The FAME structure is position-restrained, while the water molecules remain unrestrained. Third, the simulation box is heated at increasing “dewetting temperatures” (*T*_dw_ = 250–400 K) at a constant volume for 3 ns. After which, the number of water molecules within the first hydration shell (3.15 Å) of each residue is analyzed. The hydrophilic residues with the water-accessible surface area strongly interact with water to maintain their hydration with increasing *T*_dw_, while the hydrophobic residues with weakly bounded water dewet. The stronger the residue–water interaction, the higher the temperature needed to dewet the FAME surface. In other words, the dewetting temperature is conceptually related to the polymer–solvent interaction parameter (c), as increasing the *T*_dw_ weakens the interaction between water and FAME. However, instead of using a single value for a repeat unit, the interaction strength is quantified at each residue level along the peptide backbone.

**Figure 5:**
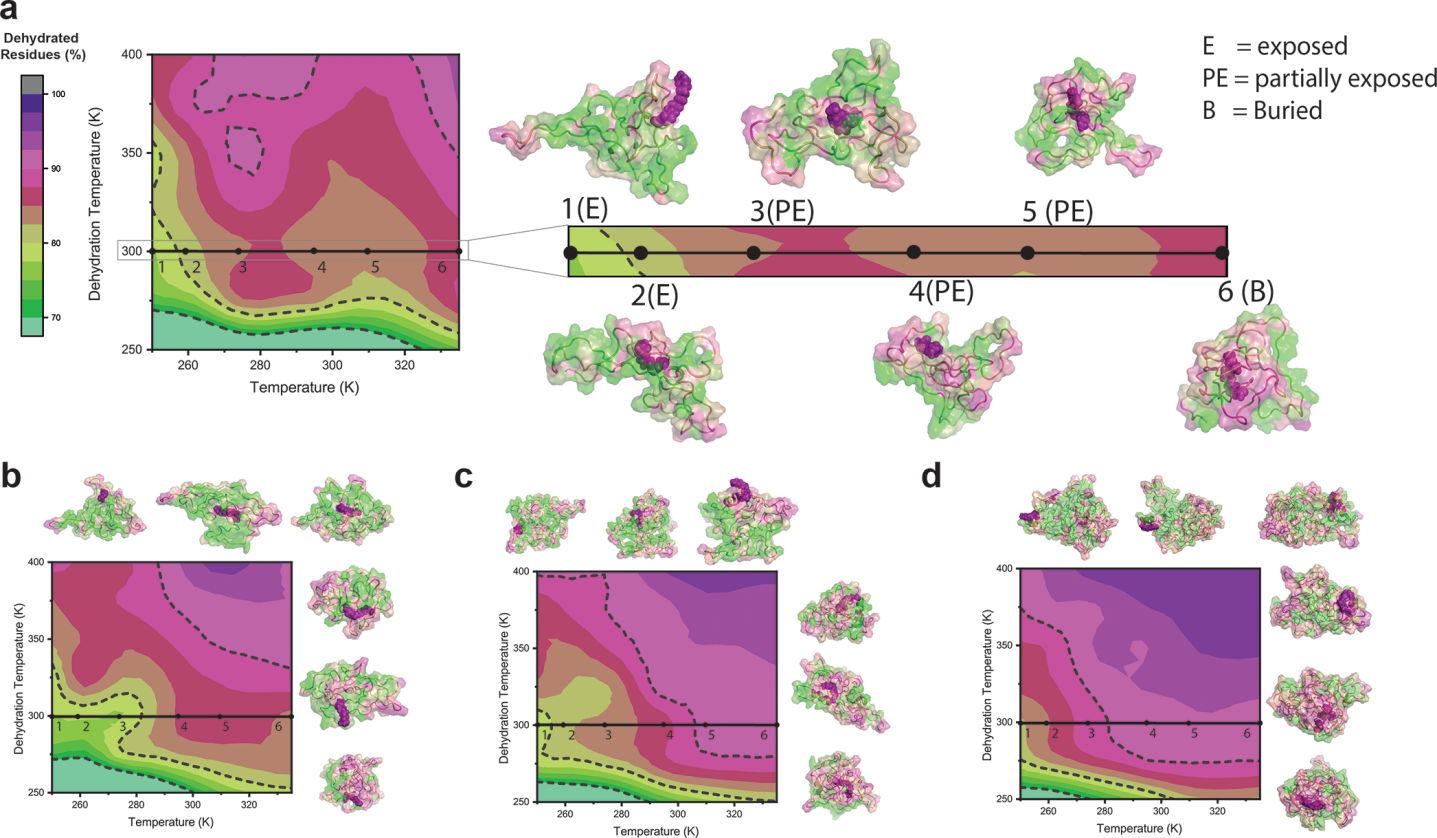
Lipidation induces hydrophobic patches in ELPs. Contour plots represent the percent dehydrated residues for each FAME as a function of simulation (*T*_MD_) and dewetting temperature (*T*_dw_). (a–d) myr-V_20_; myr-V_30_, myr-V_40_, and myr-V_60_. The horizontal line is drawn to highlight the changes in structure and hydration of each construct as the temperature increases. The protein surfaces are colored by residue according to the average number of water molecules in its hydration shell (purple = dehydrated, green = hydrated). In each construct, pentad repeats in the proximity of the lipid tails are more dehydrated compared to distal residues. Increasing the temperature alters the balance of interactions between lipid, protein, and water, leading to a structural rearrangement of the lipid tail. myr-V_20_ is used as a representative example: the water-exposed lipid tail (structures 1 and 2 (250 and 260 K)) is at first partially buried in the ELP domain, e.g., structures 3–5 (275, 295, and 310 K), but ultimately engulfed by the ELP domain (structure 6 (335 K)). A similar transition is observed in other constructs (see Figures S12–S16 for a magnified view of each panel).

The result of this analysis is presented in Figure 5, in which the contour plot provides a global view of hydration for each FAME chain in a space defined by (*T*_MD_, *T*_dw_), with the green-to-purple heat map schematically showing the dehydration of the polypeptide chain. Note that the heat map uses a normalized value (percentage of dehydrated residues) to account for variations in each construct length. As expected, each FAME is more hydrated at a lower temperature (i.e., the area close to the intersection of *T*_MD_ and *T*_dw_ is green). As the temperature is increased (i.e., when water–FAME interactions are weakened), the polypeptide chain is more dehydrated (green-to-purple transition along the *x* or *y*-axis).

At first glance, a qualitative comparison between the dehydration plots demonstrates that (despite having similar monomer chemistry), the dehydration patterns depend on the FAME length (cf. myr-V_20_ with myr-V_60_). However, as the FAME length increases, the dehydration patterns become more similar (cf. myr-V_40_ with myr-V_60_). This suggests that the N-terminal lipid alters the physicochemical properties of a segment of the ELP chain, but this effect is diluted as the chain length is increased—i.e., the GVGVP pentad distant from the lipid is more physicochemically like each other.

A closer inspection of the pattern of dehydration plots, however, reveals an intriguing paradox. To illustrate, we refer to the horizontal line drawn in Figure 5a for myr-V_20_. Increasing the temperature first results in the dehydration of the chain, e.g., 1 (250 K) → 3 (275 K); but this pattern is disrupted at higher temperatures, even suggesting that the hydration of FAMEs may partially increase above a threshold temperature, cf., 3 (275 K) → 4 (295 K), before more dehydration occurs. To understand the molecular origin of this seemingly paradoxical result, we inspected the equilibrium structure of FAMEs at these temperatures and colored each residue based on its calculated hydrophilicity (Figure 5a). These visualizations reveal an intriguing point: at any given temperature, dehydration is not uniform across the primary sequence or 3D structure of the polypeptide. Instead, a complex topographical pattern of hydrophobic patches is visible across the structure and especially in the proximity of the lipid. This suggests that proximity to the lipid dehydrates a portion of the ELP domain—rendering the pentad repeat units different from each other despite their identical chemical compositions. At low temperatures, most of the ELP chain is hydrated; and the lipid remains the most hydrophobic part of the molecule. Consequently, the interactions between the lipid and protein remain unfavorable. As temperature increases, the thermoresponsive ELP chain starts to dehydrate, rendering interactions between the lipid tail and polypeptide more favorable. These temperature-dependent interactions (between and among the lipid, protein, and water) promote the structural rearrangement of the lipid tail: The water-exposed lipid tail is initially partially buried in the ELP domain but ultimately is fully engulfed by the ELP domain at elevated temperature. This transition shields some of the GVGVP units from the hydrophobicity of the lipid tail, analogous to the behavior observed in the folding of molten globular structure (with exposed hydrophobic surfaces) into a compact folded structure. As shown in Figure 5b–d (and Figures S12–S16), this structural transition occurs in all FAMEs in the same temperature range (295–310 K), suggesting that it is dependent on the physicochemical characteristics of the lipid and the pentad repeat. We also point out that this temperature coincides with the onset of irreversible changes in the hydrodynamic size of FAME assemblies (Figure 2d).

### Experimental Characterization of FAMEs Thermoresponsive Assembly

**Figure 6:**
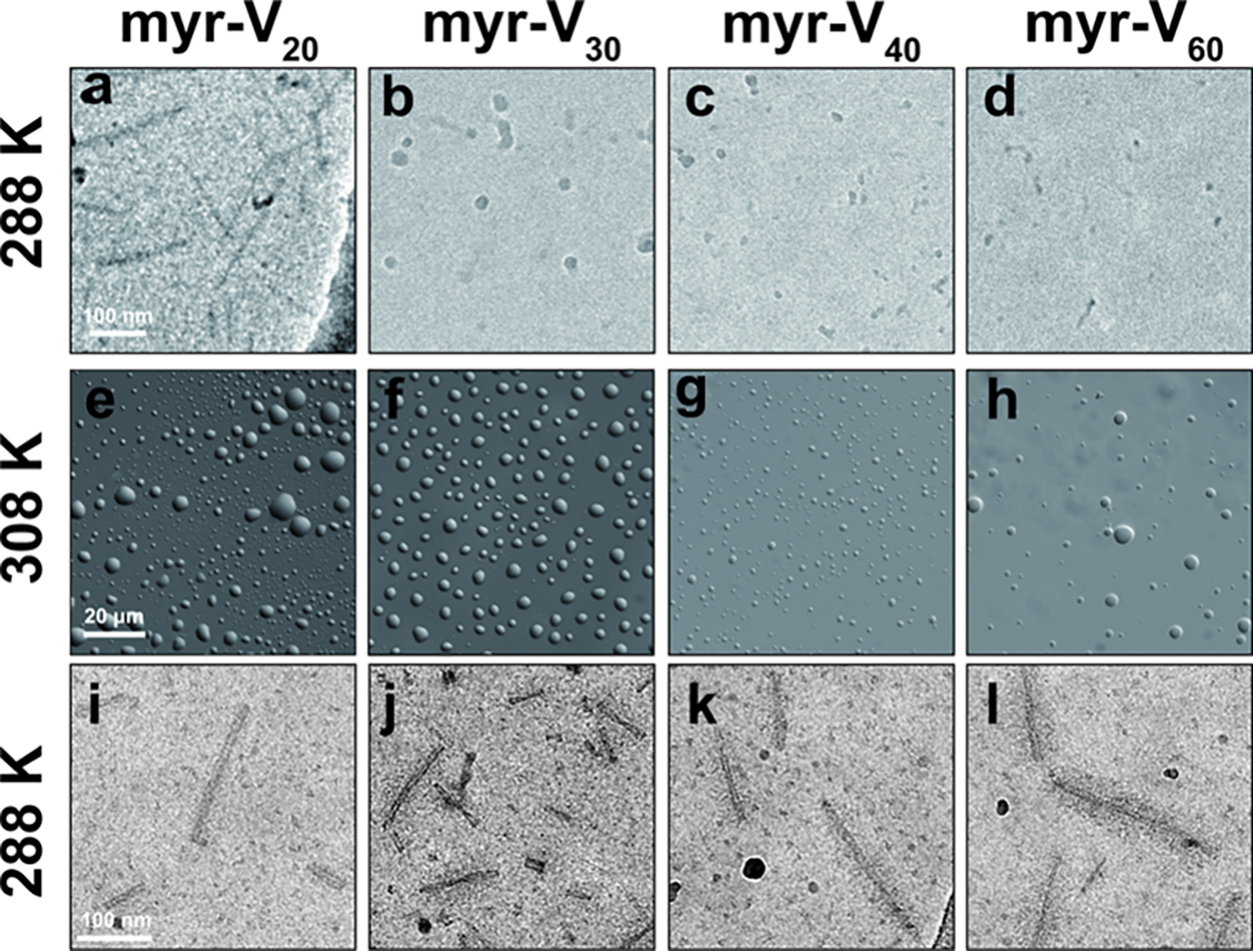
Characterization of FAME temperature-dependent assembly using microscopy. When first dissolved in cold buffer, cryo-TEM shows that myr-V_20_ (a) forms rodlike aggregates, while myr-V_30_, myr-V_40_, and myr-V_60_ (b–d) form spherical nanoparticles. (e–h) DIC microscopy confirms that at *T* = 308 K (above *T*_t_), all FAMEs undergo liquid–liquid phase separation and form micron-sized coacervates. See Figure S17 for nanoscale morphology of coacervates. (i–l) Cryo-TEM shows that upon reducing the temperature, FAMEs form anisotropic worm-like micelles with bottlebrush bristles. This structural transformation is consistent with the irreversible increase in hydrodynamic size when the FAME solution is subjected to one cycle of heating/cooling.

To investigate the temperature-dependent assembly of FAMEs across nano- and mesoscales, we used cryogenic transmission electron microscopy (cryo-TEM) and differential interference contrast (DIC) microscopy (Figure 6). When first dissolved in cold buffer (288 K), the condition corresponding to the white strip in Figure 2d, myr-V_20_ primarily formed rodlike aggregates (width = 11 ± 2 nm, length = 134 ± 51 nm). In contrast, longer FAMEs preferentially formed spherical nanostructures, with diameters inversely correlated with the length of the ELP domain: myr-V_30_ (24 ± 4 nm), myr-V_40_ (16 ± 3 nm), and myr-V_60_ (11 ± 2 nm). The discrepancy in the values reported by DLS and cryo-TEM are due to the following reasons: The high hydration of ELPs renders them almost invisible in cryo-TEM. Therefore, only a fraction of each assembly is visualized in cryo-TEM. On the other hand, the scattering profile (and correlation function) results from the interaction of light with the entire particle. However, the cumulant analysis only provides an average hydrodynamic radius and PDI. For nonspherical particles, this effective hydrodynamic radius is different from the particle length.

Because DLS suggested that FAMEs form large micron-size aggregates above their *T*_t_, we investigated the morphology of FAMEs at 308 K using both light and electron microscopy (the condition corresponding to the first red strip in Figure 2d). At this temperature, DIC confirmed the formation of liquid-like coacervates for all FAMEs, with diameters ranging from 0.5 to 8 μm. Interestingly, at this temperature, cryo-TEM reveals that all FAMEs form an extensive network of fibers (bundle) within these droplets (Figure S17), a structural feature that is far below the resolution of light microscopy but is also reported in spider silk coacervates.^81^

When the temperature is reduced to 288 K (first blue strip in Figure 2d), coacervates become completely dissolved in buffer, but cryo-TEM showed that FAME fibers are converted into discrete worm-like micelles. Unlike bundles of fibers observed above *T*_t_, these worm-like micelles are generally well separated, and their direction remains uncorrelated with each other. Despite these similarities in shape, the length of ELP has subtle effects on the features of these one-dimensional (1D) assemblies. For myr-V_40_ and myr-V_60_, the corona shows distinctive structural features of bottlebrush assemblies, while the corona of myr-V_20_ and myr-V_30_ show significantly lighter contrasts than their core. This change in nanoscale morphology is consistent with the DLS results (Figure 2d), which showed that the size of FAME constructs irreversibly increases after the first cycle of heating/cooling.

## Conclusions and Outlook

Our results reveal a detailed molecular picture of changes in FAMEs structure and hydration (and the interplay between these variables) as a function of ELP length and temperature as summarized here: (1) Increasing the FAME length promotes the lipidation site to adopt a more stable secondary structure (α-helix) while increasing the temperature denatures (melts) this structure. (2) At low temperatures, the lipid tail remains the most hydrophobic part of the molecule and is more exposed to solvent as the interactions between the lipid and polypeptide are unfavorable. This exposure to solvent may explain the propensity of FAMEs to self-assemble at low temperatures as a mechanism to reduce the solvent-accessible surface of the lipid tail. (3) More importantly, the hydrophobicity of the lipid alters the hydration pattern of nearby residues, rendering pentad with identical chemical composition to be essentially different from each other and increasing the effective size of the hydrophobic domains. (4) Finally, the simulation reveals a delicate interplay between the structure and hydration of FAMEs as a function of temperature: Increasing the temperature promotes the interaction between the lipid and dehydrated ELP chains, leading to a structural rearrangement of the lipid. This transition alters the hydration pattern of FAMEs (and their constitutive pentads) by shielding the lipid in a nascent hydrophobic core.

Although MD simulations were conducted at the single chain level, the changes to structure and hydration of FAMEs align well with the observed morphological changes. Therefore, they can provide critical insights into the molecular mechanism of thermoresponsive assembly of FAMEs. The ELP domains are hydrophilic at low temperatures, and their overall interactions remain repulsive due to the volume exclusion effect. However, lipid-induced dehydration alters the effective hydrophilic volume fractions (*f*) so that even myr-V_20_ (with theoretical *f* = 0.98) forms rodlike aggregates, which are expected when 0.3 < *f* < 0.5. In contrast, the other FAMEs with longer lengths (and reduced hydrophobicity) form spherical particles when first dissolved in the buffer. Intriguingly, myr-V_20_ also contains a disordered and dynamic lipidation site, which allows a higher packing density of chains required for rod formation. Conversely, the rigid structure of the α helices found in other FAMEs should reduce packing efficiency, thus favoring the formation of spherical nanoparticles.

Increasing the temperature dehydrates the thermoresponsive ELP chains and renders the lipid–ELP interactions favorable, promoting the structural transition of the lipid tail (exposed-to-buried). The association of the lipid with ELP further dehydrates segments of the corona, effectively increasing the size of hydrophobic domains. Consequently, the packing parameter is increased, favoring the formation of rodlike (1D) assemblies.

We point out that increasing the temperature also increases ELP–ELP interactions and promotes their liquid–liquid phase separation to form coacervates. The coacervation can significantly increase the local concentration of FAMEs and promote the formation of long fibers shown in Figure S17. Reducing the temperature *T*_t_ rehydrates the ELP chains become rehydrated, which can provide steric stabilization necessary to break the fibers into smaller worm-like micelles.

This work demonstrates a molecular mechanism to explain how temperature-dependent properties of FAMEs are determined by an interplay between various domains/fragments of these macro-amphiphiles. In the five constructs studied, ELP length altered the propensity of the lipidation site to adopt a stable secondary structure. We show that proximity of the lipid to ELPs results in different patterns of hydrophobicity among sequences of pentapeptides that vary only in numbers of repeats. And finally, the structure of the lipidation site and the proximity (and/or interaction) between the ELP and lipid are temperature-dependent, which should allow for fine-tuning of the amphiphilic ratio of the FAME molecules. This work provides the molecular-level understanding of factors than govern the structural transition of FAME ensembles and therefore contributes to the design principles essential for the next generation of smart amphiphiles.

Here, we used MD simulations to study the emergence of hydration heterogeneities and to provide an atomistic view of how temperature alters these hydrophilic/hydrophobic patches. Molecular dynamics simulations are increasingly being utilized to not only provide molecular-level insights that are unattainable experimentally but also explain dynamical behavior observed in self-assembled nanostructures.^82^ While other work has recently used a conceptually similar methodology to predict protein interaction interfaces,^83,84^ solvation remains a dark corner for the design of soft materials.^85−87^ Compared to synthetic amphiphiles and polymers, this problem has been particularly intractable for peptide- and protein-based materials as their complex chemical structure interferes with the full accounting of their interactions with water. Our work extends the use of these computational methodologies to reveal how PTMs can introduce emergent nanoscale heterogeneities in the hydration pattern of self-assembled structures, suggesting the applicability of our approach to other systems such as peptoids,^88,89^ peptides,^90,91^ and polymers.^92−94^

Our integrated approach may also shed light on the biophysical origins of the numerous myristoylation sites (>5000 confirmed or predicted sequences) in eukaryotes, fungi, and viruses.^95^ Despite the enhanced bioinformatics tools available to identify these motifs, our understanding of how lipidation alters the structure and assembly of these sites remains limited. This dearth of structural information is due in part to difficulties in synthesizing proteins with compositionally defined lipidation patterns. Even when the lipidated protein can be accessed at quantities sufficient for structural biology purposes, other technical challenges (e.g., peak broadening in NMR due to intermolecular association of proteins) may hinder obtaining high-resolution structural information for the lipidation site.^96^ This lacuna limits our understanding of the energetic interplay and biomaterial consequences of these lipidation sites. In principle, the nanoassembly of FAMEs (which is analogous to the quaternary organization of proteins) can be used to compare the properties of various (non-)canonical lipidation sites. Therefore, our integrated approach provides a framework to explore this untapped design space to synthesize hybrid nanoassemblies with programmable structure, function, and capabilities.

Building on the foundation of this study, our future work will use multichain simulations to capture the assembly process of FAMEs and to quantify how the assembly process may alter the structure and hydration of each chain. Moreover, we envision revealing how the nanoscale assembly of FAMEs influences the formation and material properties of their coacervates. Finally, (de)protonation of histidine residues in the lipidation site may provide an avenue to encode pH-responsiveness in FAME assemblies for biomedical application. These studies are ongoing in our laboratories and will be reported in due course.

## Supporting Information Available

Sequence of primers, plasmid information, MALDI spectra, temperature-dependent hydration data, characterization of FAME library using gel electrophoresis, MALDI-TOF-MS, RP-HPLC, dynamic light scattering, TEM, and simulation data showing the temperature-dependent alteration in the structure and hydration of FAMEs (PDF)
