# Supplementary material for: Lipidation Alters the Structure and Hydration of Myristoylated Intrinsically Disordered Proteins
Source: Biomacromolecules. 2023 Feb 9;24(3):1244–57. doi: 10.1021/acs.biomac.2c01309 (PMC10017028; doi:10.1021/acs.biomac.2c01309)
Supplement: Supplementary file 1 — bm2c01309_si_001.pdf [file bm2c01309_si_001.pdf]

Lipidation Alters the Structure and Hydration of Myristoylated Intrinsically Disordered Proteins

Jingjing Ji,[‡] Md Shahadat Hossain,[‡] Emily N. Krueger, Zhe Zhang, Shivangi Nangia, Britnie Carpentier, Mae Martel, Shikha Nangia,* and Davoud Mozhdehi*

‡ These authors contributed equally.

*Corresponding authors. Email: snangia@syr.edu and dmozhdeh@syr.edu

Supplementary Information

Table of Contents

1. Supplementary Tables S2
2. Supplementary Figures S6
3. References S29

1. Supplementary Tables

Table S1. PCR primers used in this study

Primer name	Sequence	Used for	Optimized annealing Temperature (°C)
ΔHis-FWD	5′-AAGGACCACAAATTTTGGCG-3′	Deletion of (6xhis) from pETDuet-1_NMT_rs	63
ΔHis-REV	5′-GCTGCTGCCCATGGTATA-3′		
V30-FWD	5′-GGAGTAGGTGTCCCTGGA-3′	Mutagenesis of Addgene plasmid # 67014 to remove the leader sequence (Δ2-9) (6xhis-SSG)	56
V30-REV	5′-CATATGTACTCCTCCTTCTTAAAGTTAAAC-3′		

Table S2. Plasmids used in this study

Constructs	Vectors used[a]	Important features of the vector
myr-V_{20}	pETDuet-1_NMT_rs-V_{20}	
myr-V_{30}	pETDuet-1_NMT_rs-V_{30}	
myr-V_{40}	pETDuet-1_NMT_rs-V_{40}	Amp^r, pBR322 Ori, bicistronic T7 promotors, LacI
Myr-V_{50}	pETDuet-1_NMT_rs-V_{50}	
myr-V_{60}	pETDuet-1_NMT_rs-V_{60}	

a. NMT= GSS(Δ1-35) yeast NMT (accession # P14743); rs = GLYASKLFSNLGHHHHHHHH; V_x = $(GVGVP)_x$GY.

Table S3. Theoretical molar mass and observed m/z of constructs in this study

Constructs	Molecular Weight, Da (average)[a]	Observed $[M+H]^+$	Δ (%)
myr-V_{20}	10986.32	10989.53	0.02
myr-V_{30}	15081.40	15078.08	-0.03
myr-V_{40}	19176.62	19181.46	0.02
myr-V_{50}	23391.84	23369.21	-0.1
myr-V_{60}	27365.96	27283.49	-0.31

a. molecular weight of each construct without the initial formyl methionine + 228.38 (myristic acid) – 18.01 (H_2O)

Table S4. The slope, Y-intercept, and goodness-of-fit for the best linear fit of length-normalized N_w and N_{pp} as the function of temperature

Constructs	N_w/L vs. Temp			N_{pp}/L vs. Temp		
	Slope [90% Confidence Interval]	Y-intercept [90% Confidence Interval]	R^2	Slope [90% Confidence Interval]	Y-intercept [90% Confidence Interval]	R^2
myr-V_{20}	-0.08 [-0.09, -0.07]	46.78 [42.97, 50.60]	0.98	0.03 [0.02, 0.04]	3.73 [1.86, 5.56]	0.96
myr-V_{30}	-0.13 [-0.16, -0.10]	59.96 [50.82, 69.10]	0.95	0.10 [0.05, 0.17]	-14.00 [-25.83, -2.16]	0.86
myr-V_{40}	-0.11 [-0.13, -0.08]	51.32 [43.71, 58.93]	0.95	0.06 [0.04, 0.09]	-3.09 [-10.20, 4.02]	0.88
Myr-V_{50}	-0.07 [-0.08, -0.06]	40.59 [36.90,44.29]	0.97	0.04 [0.03, 0.05]	5.79 [2.53-8.89]	0.93
myr-V_{60}	-0.05 [-0.07, -0.03]	32.74 [27.27, 38.20]	0.89	0.02 [0.00, 0.04]	12.03 [5.83, 18.23]	0.55

2. Supplementary Figures

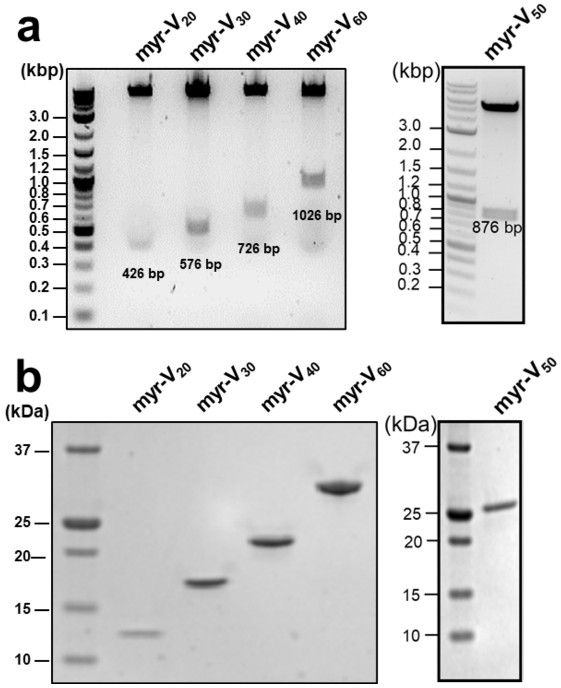

Figure S1. Characterization of FAMEs library. a) An agarose gel is used to analyze the length of DNA fragments after restriction mapping of FAME plasmids (Table S2) using *NdeI* and *XhoI*. In each channel, the higher molecular weight band corresponds to the plasmid backbone (+ NMT), and the smaller fragment is the coding sequence of FAMEs. **b)** SDS-PAGE is used to characterize the purified FAMEs. The protein bands are stained with SimplyBlue for visualization.

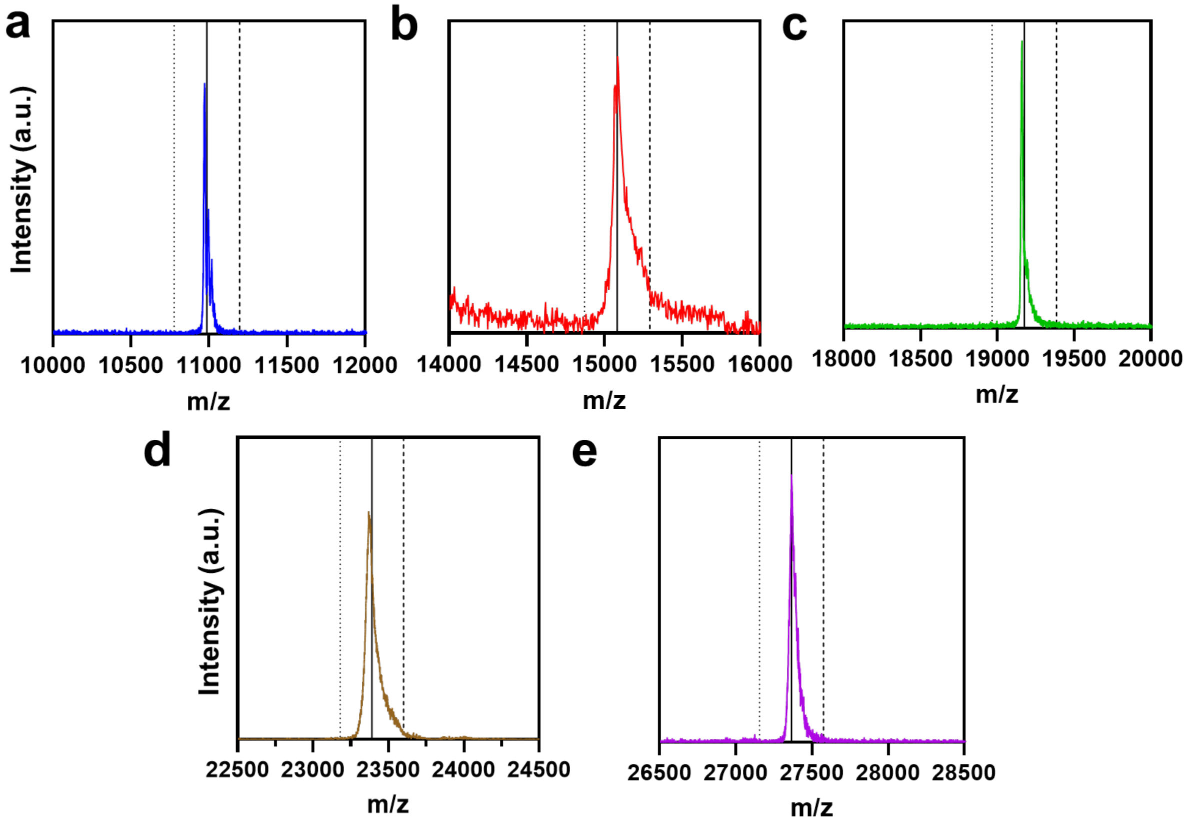

Figure S2. MALDI-TOF-MS analysis of full-length constructs is consistent with the modification of FAMEs with a single myristoyl group. **a)** myr-V_{20}; **b)** myr-V_{30}; **c)** myr-V_{40}; **d)** myr-V_{50}; e) myr-V_{60}. In each panel, the vertical lines correspond to the average theoretical mass ($[M+H]^+$) of various isoforms: unmodified (dotted); modified with a one (solid) or two (dashed) myristoyl group(s). See the supplementary note after Figure S3.

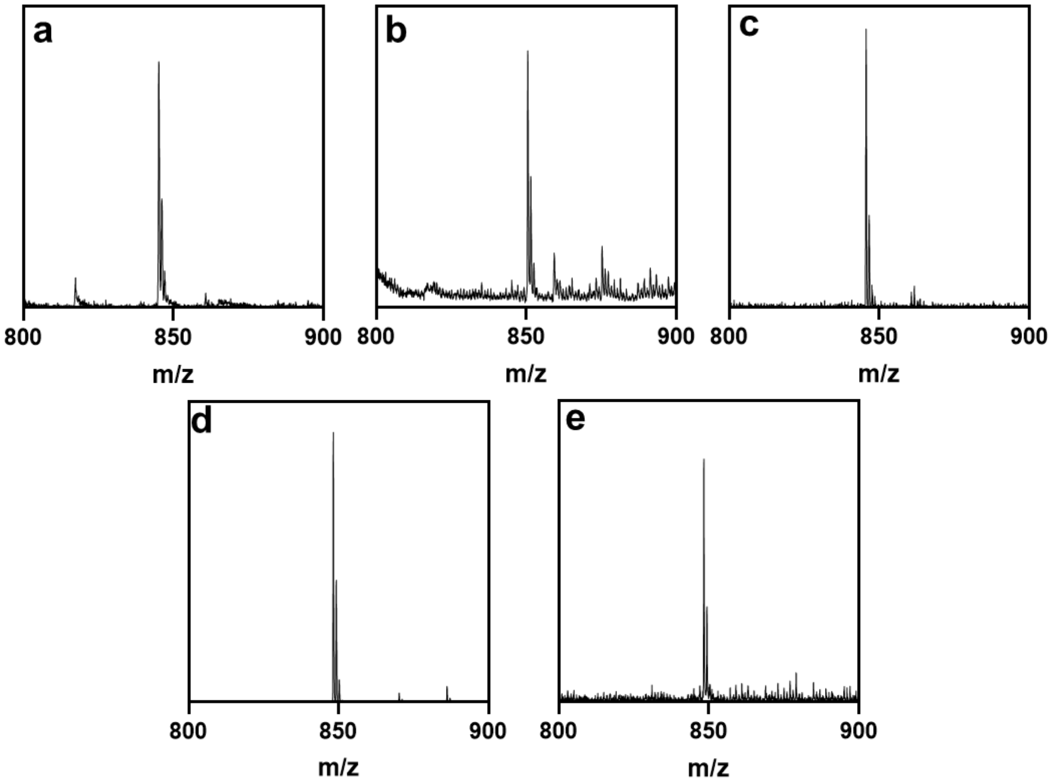

Figure S3. MALDI-TOF-MS confirms the N-terminal myristoylation of FAMEs. MALDI-TOF spectra for a) myr-V_{20}, b) myr-V_{30}, c) myr-V_{40}, d) myr-V_{50}, and e) myr-V_{60} after trypsin digestion. The observed m/z of ~849-850 Da agrees with the expected mass of myristoylated N-terminus fragment GLYASK ($[M+H]^+$= 849.5508 Da).

Note. We are cognizant that MALDI- MS is not quantitative and therefore have relied on both RP-HPLC and MALDI-TOF-MS to establish the purity and identity of each construct. Our previous work has shown that the retention time of ELPs in RP-HPLC increases as the number of appended lipids increase.[1] In this paper, we observe only one peak with a retention time longer than unmodified proteins. When this peak is subjected to MALDI-TOF, the increase in the observed molecular weight corresponds to the addition of a single myristoyl group (Figure S2). In addition to the N-terminal glycine, the only other nucleophilic site on each FAME is a lysine residue in the lipidation site. However, NMT is highly selective toward modifying the N-terminal Glycine residue.[2] Trypsin digest (Figure S3) confirms that this lysine residue is unmodified (as acylation of lysine will inhibit proteolytic digest), narrowing down the site of the lipidation to the N-terminal glycine residue.

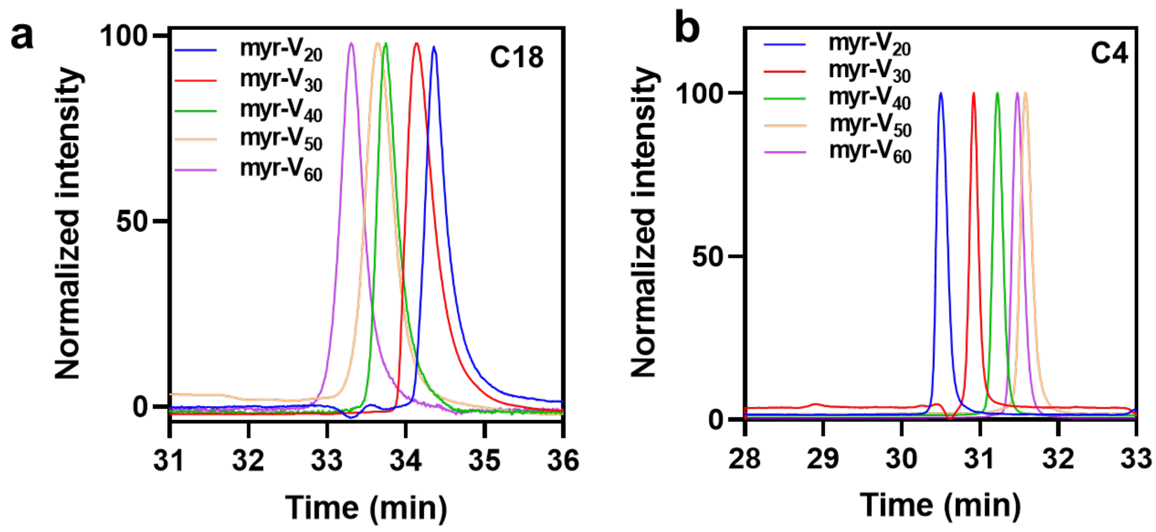

Figure S4. RP-HPLC analysis of FAMEs using **a)** C18-functionalized and **b)** C4-functionlized columns.

<u>Note.</u> Retention times in RP-HPLC are determined by a balance of complex interactions between the analyte, eluent, and immobilized phase.[3] We suggest that the myristoyl group preferentially interacts with the hydrophobic coating of C18-functionalized columns. Under this interaction mode, increasing the length of the ELP reduces the ability of the lipid to interact with the column, thus reducing the residence and elution times. To support this assertion, we analyzed the FAME library using a C4-functionalized column, expecting that the shorter length of hydrocarbon would alter the balance of interactions between FAME and the beads. Consistently, we observe a different elution pattern: The retention time of FAMEs generally increases with ELP length—myr-V_{20} (30.5 min), myr-V_{30} (30.9 min), and myr-V_{40} (31.2 min). However, the observed elution times of myr-V_{50} and myr-V_{60} are reversed, i.e., 31.6 and 31.5 min.

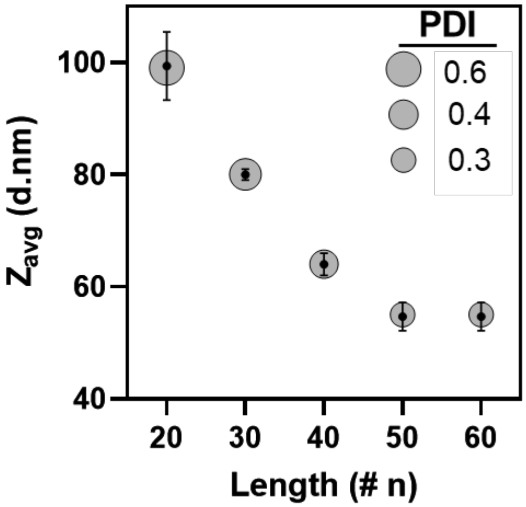

Figure S5. The hydrodynamic radii and polydispersity index (PDI) of FAMEs decrease with ELP length. DLS is used to study the hydrodynamic size of FAME assemblies when first dissolved in PBS at 288 K. The autocorrelation functions are fit to the cumulants method to derive the hydrodynamic radius (the symbol and error bar) and PDI (depicted using the bubble area). Error bars are the standard deviations of three measurements.

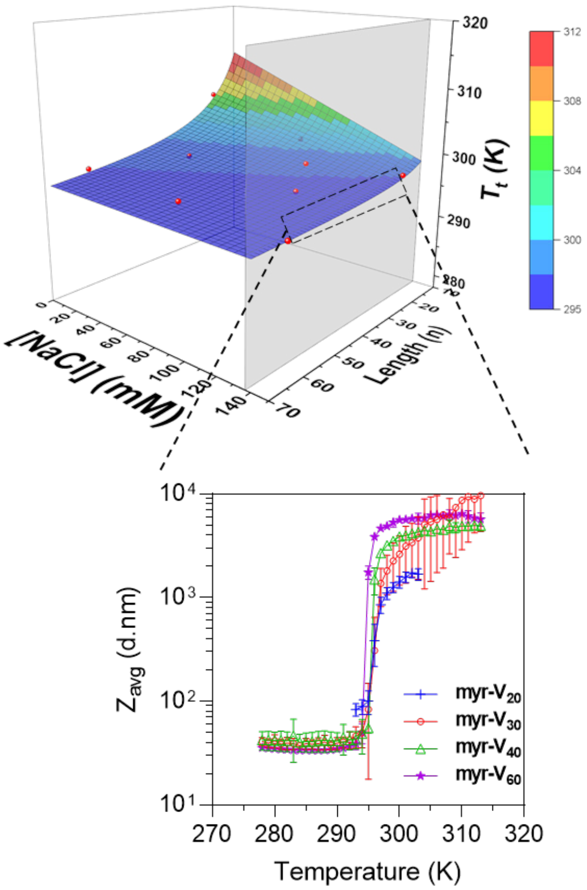

Figure S6. Dependence of the transition temperature (T_t) of FAMEs on their length and salt concentration. The Design Expert® software was used to design a series of experiments to determine the dependence of FAMEs T_t on ELP length and salt concentration [NaCl]. In each case, protein solutions (2.5 μM in phosphate buffer with indicated salt concentration) were analyzed by DLS at 278 – 315 K at 1 K increments to identify the threshold temperature above which the hydrodynamic size of FAMEs is increased significantly (shown as red spheres in the contour plot). By fitting these data, we derived an empirical equation describing the dependence of T_t on FAME length (L) and [NaCl]: $T_t = 20.4 + 229.4/L - [NaCl] \times (1.3/L - 0.01)$, plotted as a surface function. To confirm this prediction, we then empirically determined the T_t of four FAMEs in PBS using the same method, as shown in the extracted plot. Unlike nonlipidated ELPs, the transition temperature of this FAME library does not vary strongly with length, agreeing with the prediction of the empirical model.

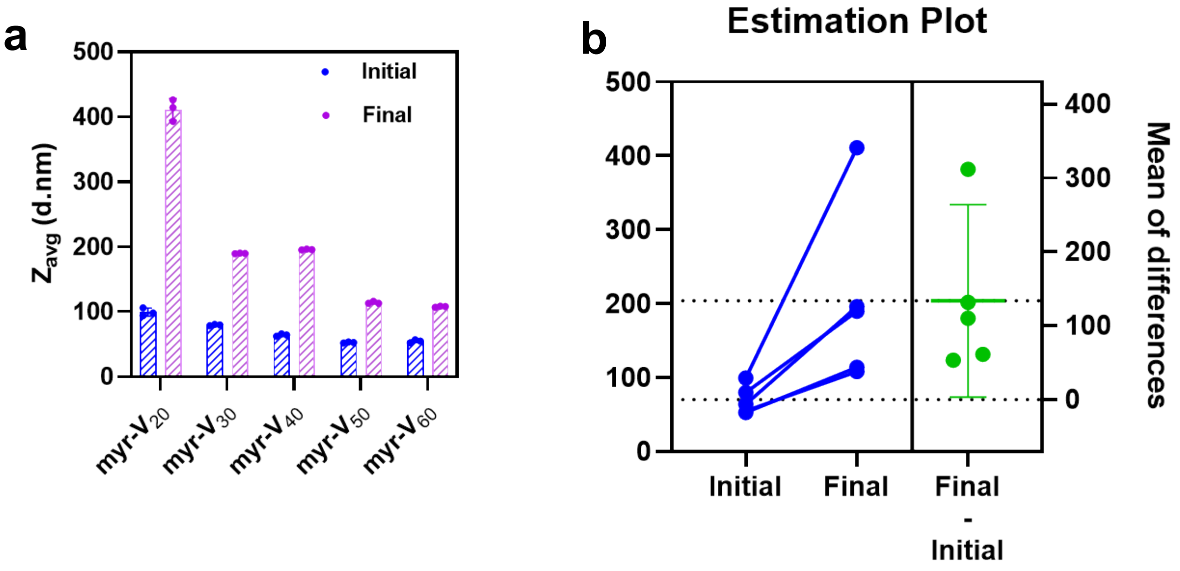

Figure S7. The size of FAME assemblies increases irreversibly upon heating and cooling the solution. a) The average hydrodynamic diameter of each construct at 288 K, determined from a cumulant fit of DLS autocorrelation functions. Blue bars show the average size when FAMEs are just dissolved in cold buffer, and purple bars show the size after three cycles of heating/cooling above the transition temperature. FAMEs hydrodynamic size increases after thermal treatment (paired two-tailed t-test, $t(4) = 2.85$, $p = 0.046$). **b)** The estimation plot displays the magnitude of this irreversible increase in size.

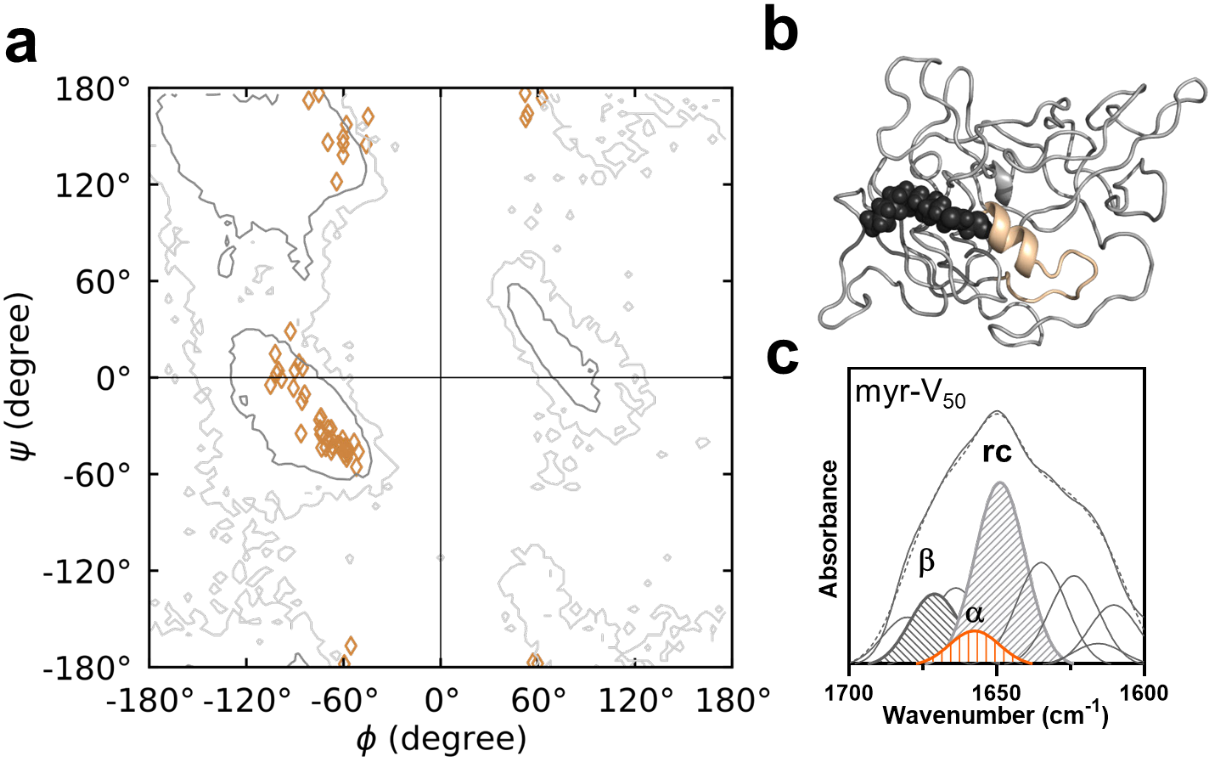

Figure S8. ELP length alters the propensity of the lipidation site to adopt a stable secondary structure. a) Ramachandran plot (ϕ, ψ dihedral angle distributions) for the backbone residues in the lipidation site in M-V_{50} (wheat ◊) at 295 K over last 1 µs of the MD simulation. **b)** Snapshot of a representative structure at 295 K. The lipidation site adopts a well-defined right-handed alpha helix. **c)** Consistent with MD simulations, deconvoluted FT-IR spectra of M-V_{50} (100 µM at in D_2O) exhibit a peak at 1657 cm^{-1}, attributed to α-helices in a deuterated environment.

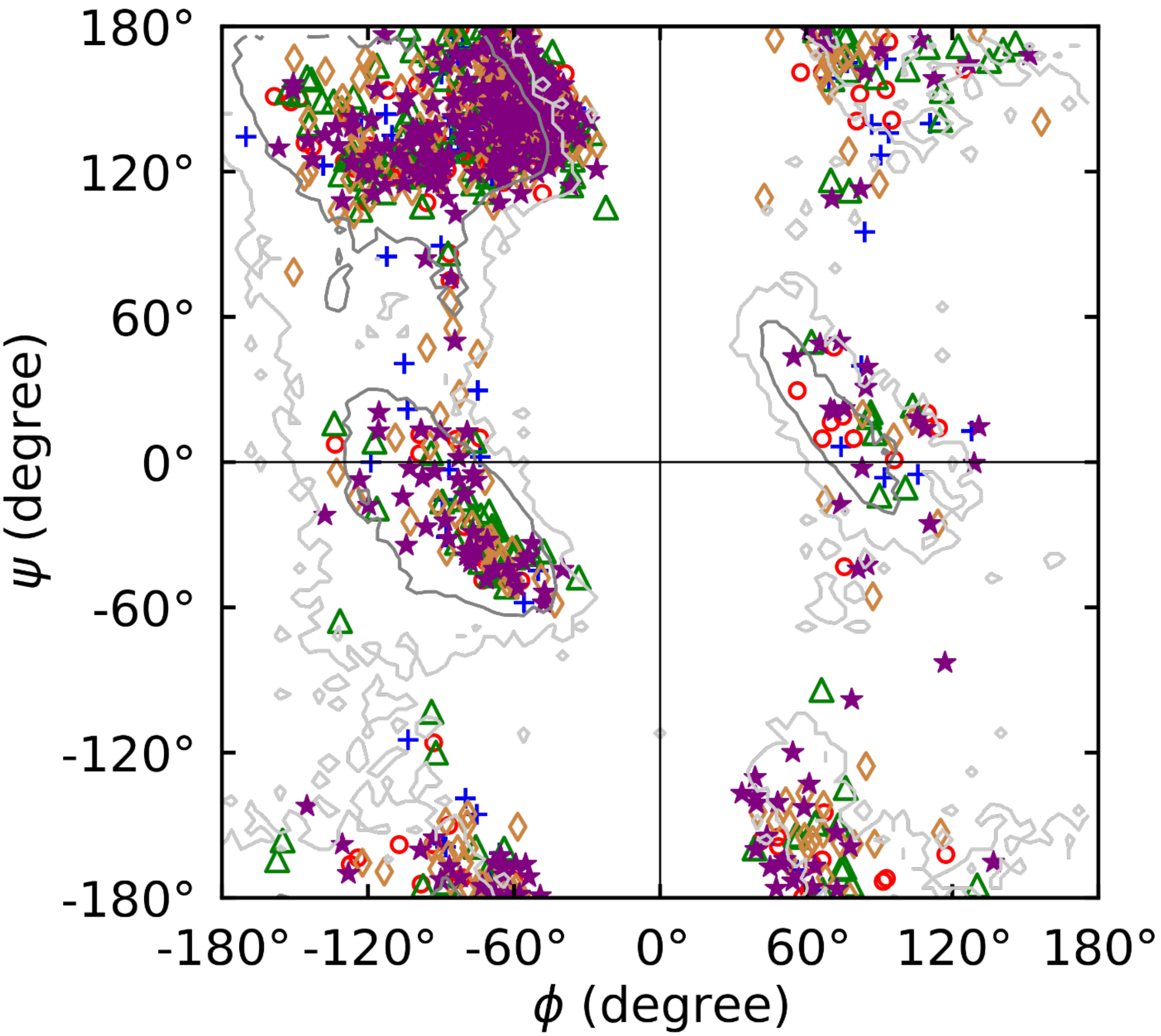

Figure S9. Lipidation does not change the global structure of ELP-residues. Ramachandran plots (ϕ, ψ dihedral angle distributions) for the backbone residue VPGVG motifs in myr-V_{20} (blue +), myr-V_{30} (red o), myr-V_{40} (green Δ), myr-V_{50} (wheat ◊), and myr-V_{60} (purple *) at 295 K. The dispersion of data points over the conformational space shows that the ELP domains remained highly disordered throughout the simulation.

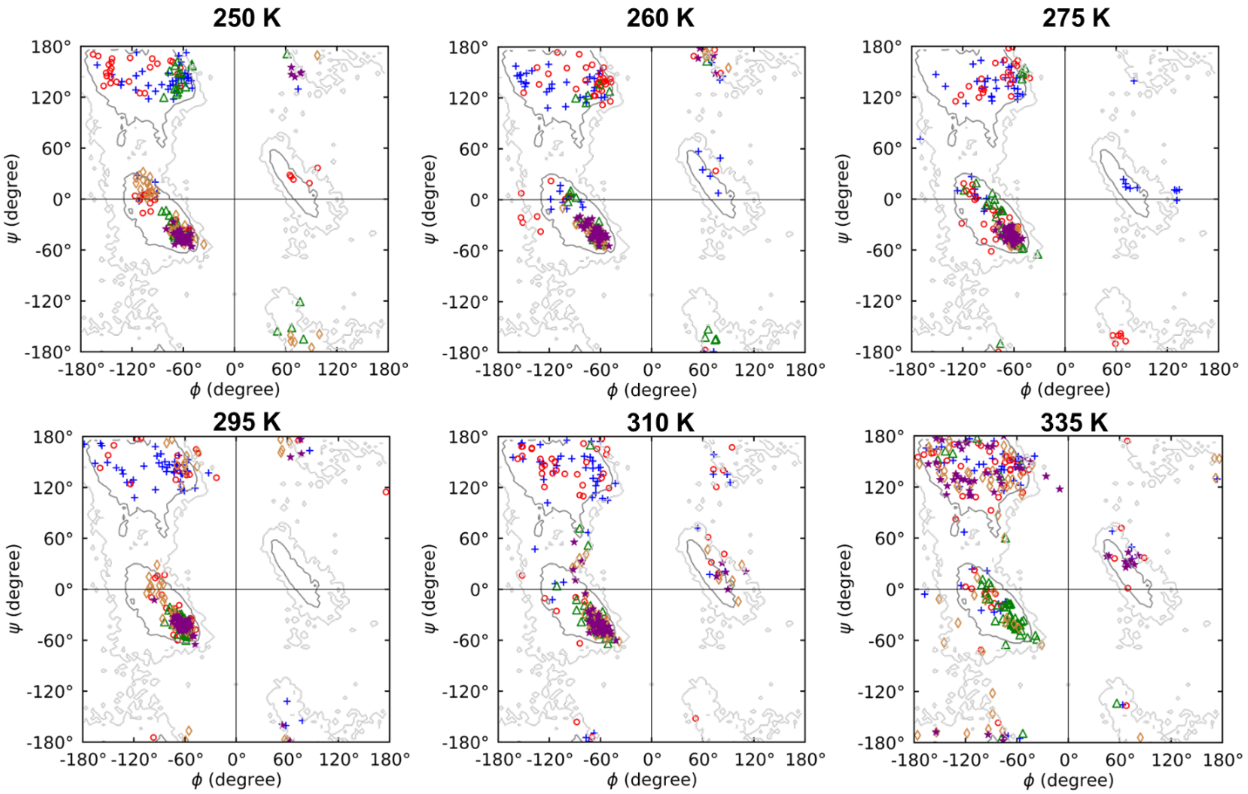

Figure S10. Increasing temperature melts the secondary structure of the lipidation site. Ramachandran plots (ϕ, ψ dihedral angle distributions) for the backbone residues in the lipidation site in myr-V_{20} (blue +), myr-V_{30} (red o), myr-V_{40} (green Δ), myr-V_{50} (wheat ◊), and myr-V_{60} (purple *), at 295 K over last 1 μs of the MD simulation at 250, 260, 275, 295, 310, and 335 K. The (un)folding of the secondary structure is due to the balance of enthalpy and entropy; the folded state is stabilized by hydrogen bonds, while the disordered state has higher entropy. At higher temperatures, the balance is tipped in favor of the entropy, resulting in destabilization and denaturation of the structure.

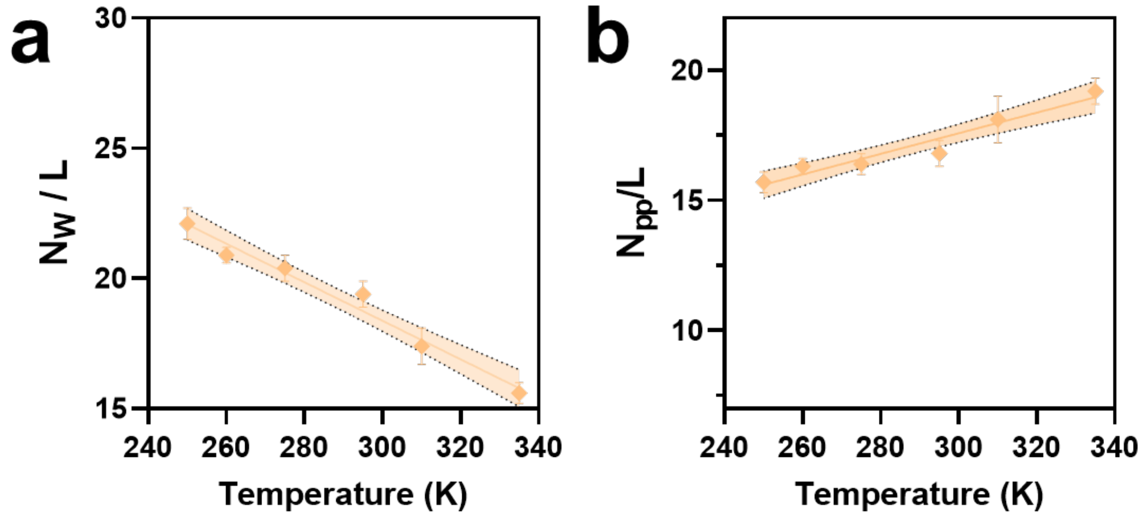

Figure S11. Temperature-dependent changes in hydration and intramolecular contact of myr-V50. **a)** The length-normalized number of water molecules (N_w) within the first hydration shell of peptide backbone vs. temperature. **b)** The length-normalized number of intrachain (peptide–peptide) contacts (N_{pp}) versus temperature **b).** The error bars represent the standard deviation calculated from the time average of simulations, and the dotted lines represent the 90% confidence interval of the fitted line. See Table S4 for the slope, Y-intercept, and R2 for each line.

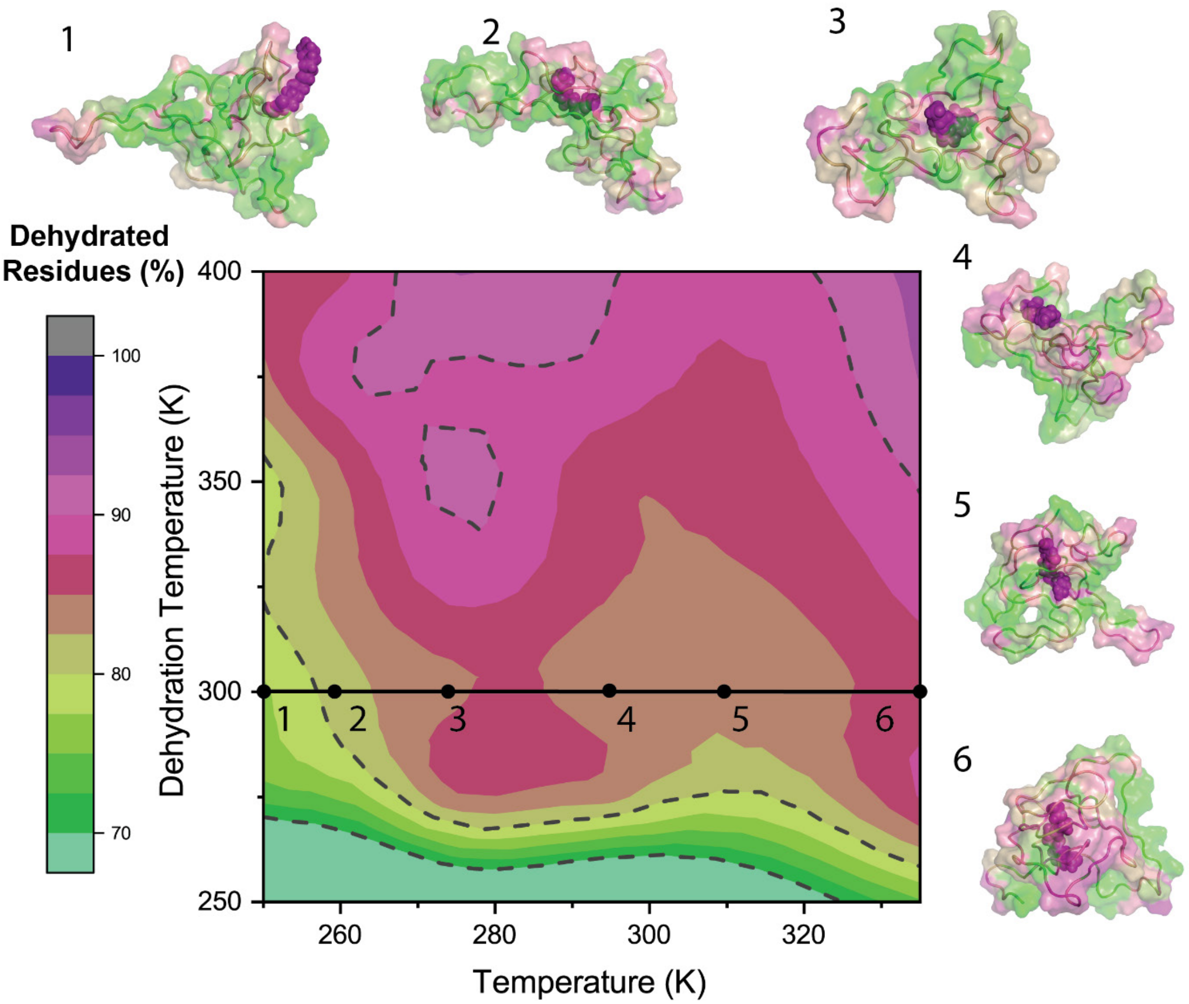

Figure S12. Lipidation induces temperature-dependent hydrophobic patches in myr-V_{20}. Contour plots represent the percent dehydrated residues for each FAME as a function of simulation (T_{MD}) and dewetting temperature (T_{dw}). The horizontal line is drawn to highlight the changes in structure and hydration of each construct as the temperature increases. The protein surfaces are colored by residue according to the average number of water molecules in its hydration shell (purple = dehydrated, green = hydrated). Pentad repeats in the proximity of the lipid tails are more dehydrated compared to distal residues. Increasing the temperature alters the balance of interactions between lipid, protein, and water, leading to a structural rearrangement of the lipid tail.

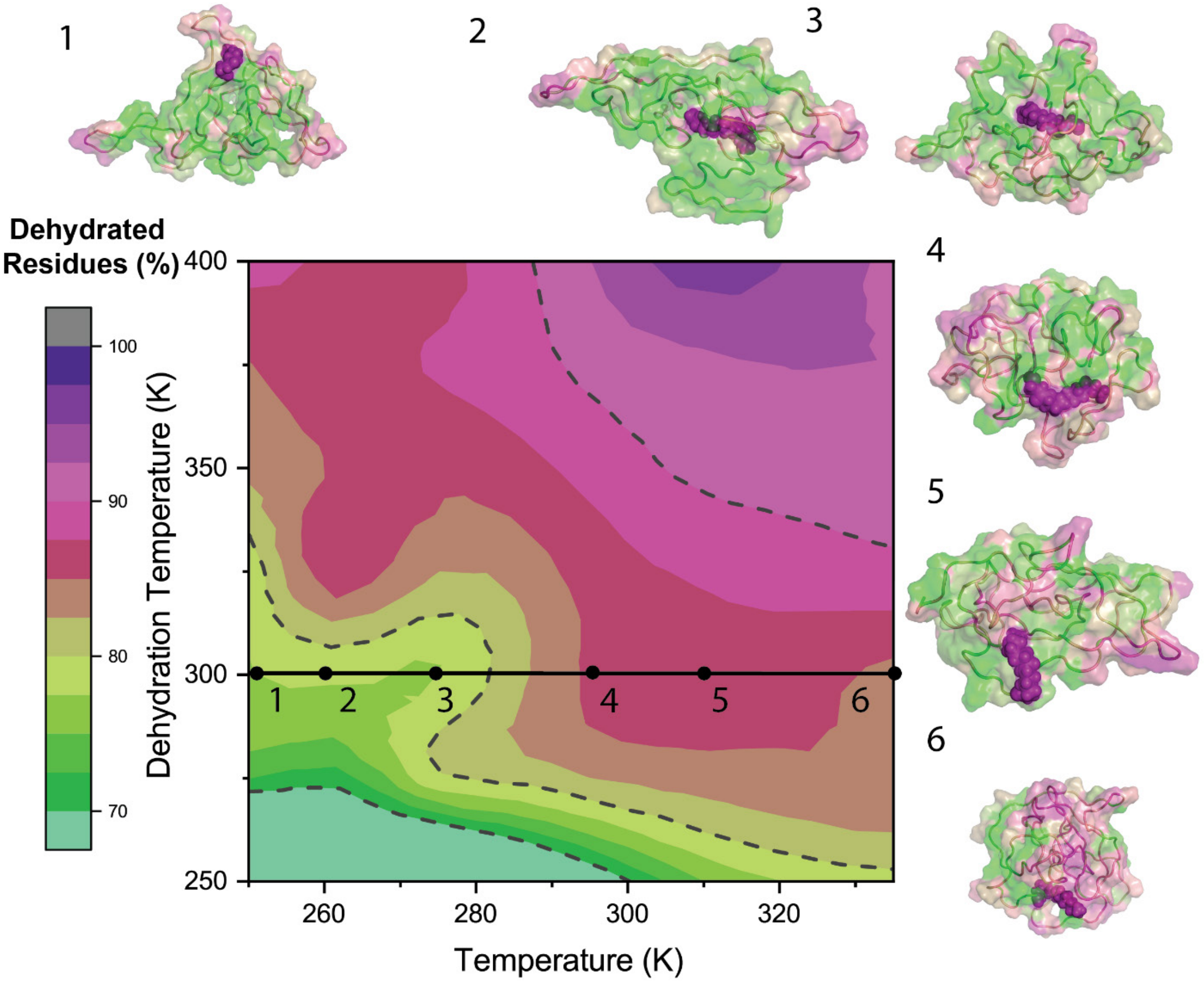

Figure S13. Lipidation induces temperature-dependent hydrophobic patches in myr-V_{30}. Contour plots represent the percent dehydrated residues for each FAME as a function of simulation (T_{MD}) and dewetting temperature (T_{dw}). The horizontal line is drawn to highlight the changes in structure and hydration of each construct as the temperature is increased. The protein surfaces are colored by residue according to the average number of water molecules in its hydration shell (purple = dehydrated, green = hydrated). Pentad repeats in the proximity of the lipid tails are more dehydrated compared to distal residues. Increasing the temperature alters the balance of interactions between lipid, protein, and water, leading to a structural rearrangement of the lipid tail.

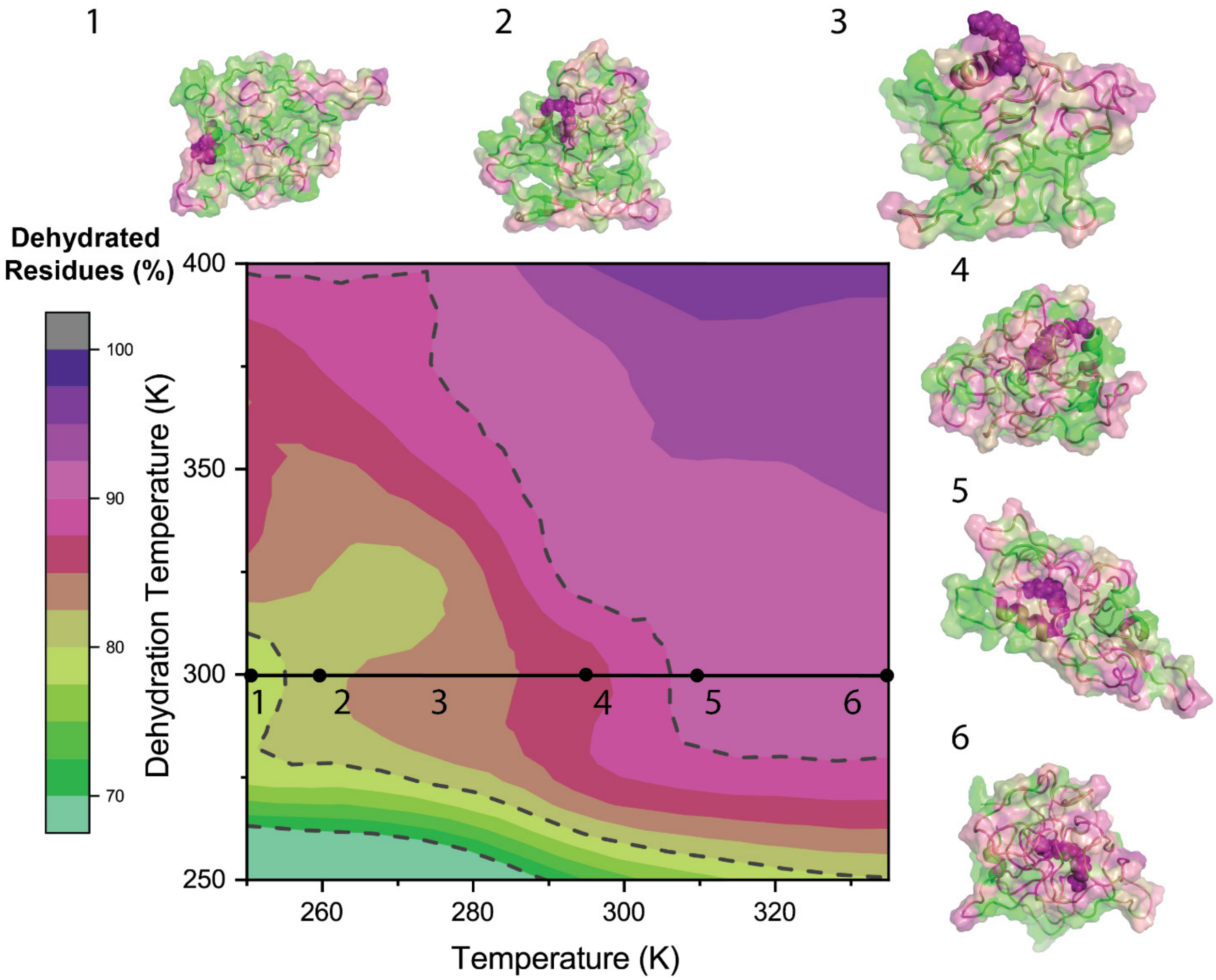

Figure S14. Lipidation induces temperature-dependent hydrophobic patches in myr-V_{40}. Contour plots represent the percent dehydrated residues for each FAME as a function of simulation (T_{MD}) and dewetting temperature (T_{dw}). The horizontal line is drawn to highlight the changes in structure and hydration of each construct as the temperature is increased. The protein surfaces are colored by residue according to the average number of water molecules in its hydration shell (purple = dehydrated, green = hydrated). Pentad repeats in the proximity of the lipid tails are more dehydrated compared to distal residues. Increasing the temperature alters the balance of interactions between lipid, protein, and water, leading to a structural rearrangement of the lipid tail.

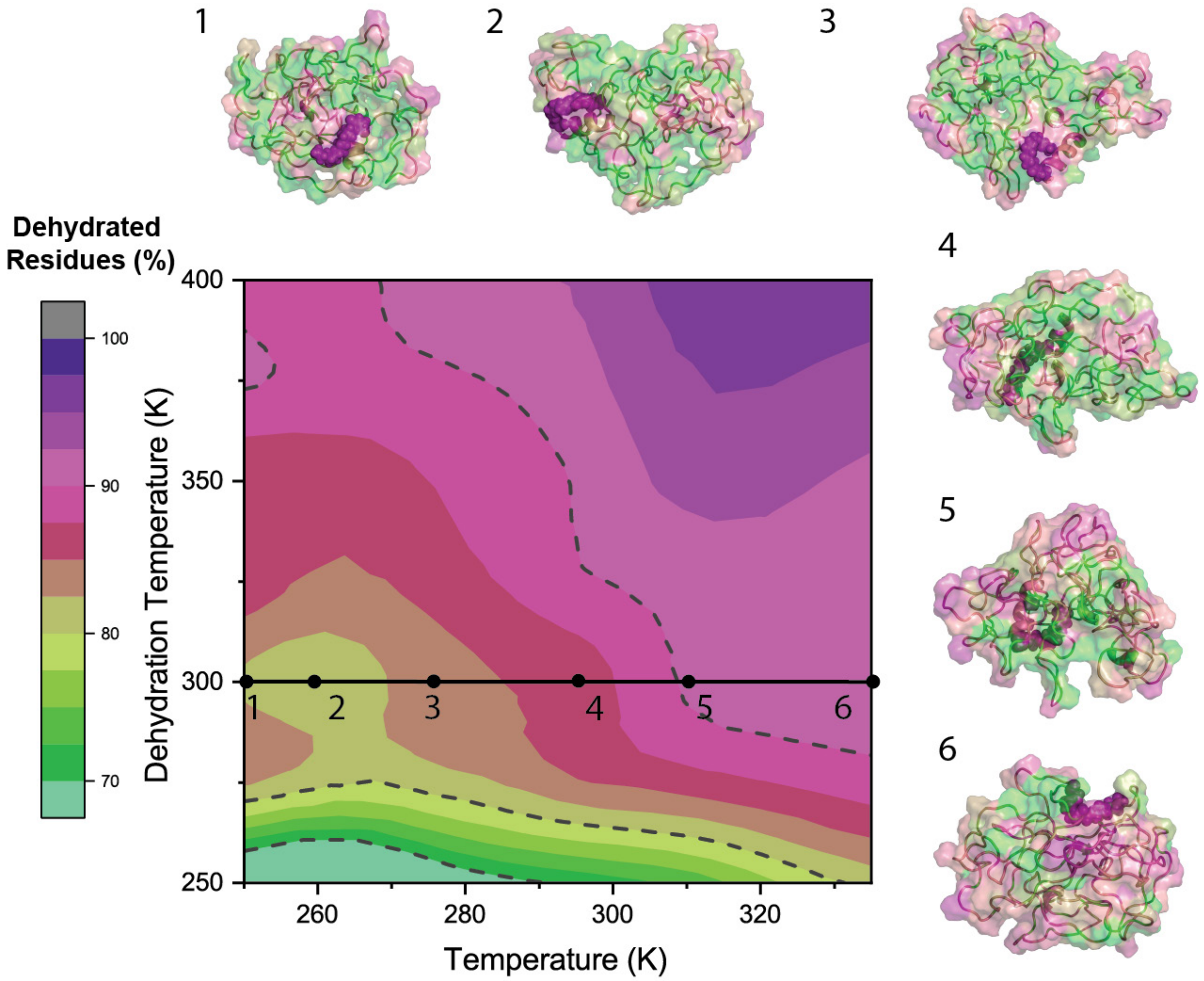

Figure S15. Lipidation induces temperature-dependent hydrophobic patches in myr-V_{50}. Contour plots represent the percent dehydrated residues for each FAME as a function of simulation (T_{MD}) and dewetting temperature (T_{dw}). The horizontal line is drawn to highlight the changes in structure and hydration of each construct as the temperature is increased. The protein surfaces are colored by residue according to the average number of water molecules in its hydration shell (purple = dehydrated, green = hydrated). Pentad repeats in the proximity of the lipid tails are more dehydrated compared to distal residues. Increasing the temperature alters the balance of interactions between lipid, protein, and water, leading to a structural rearrangement of the lipid tail.

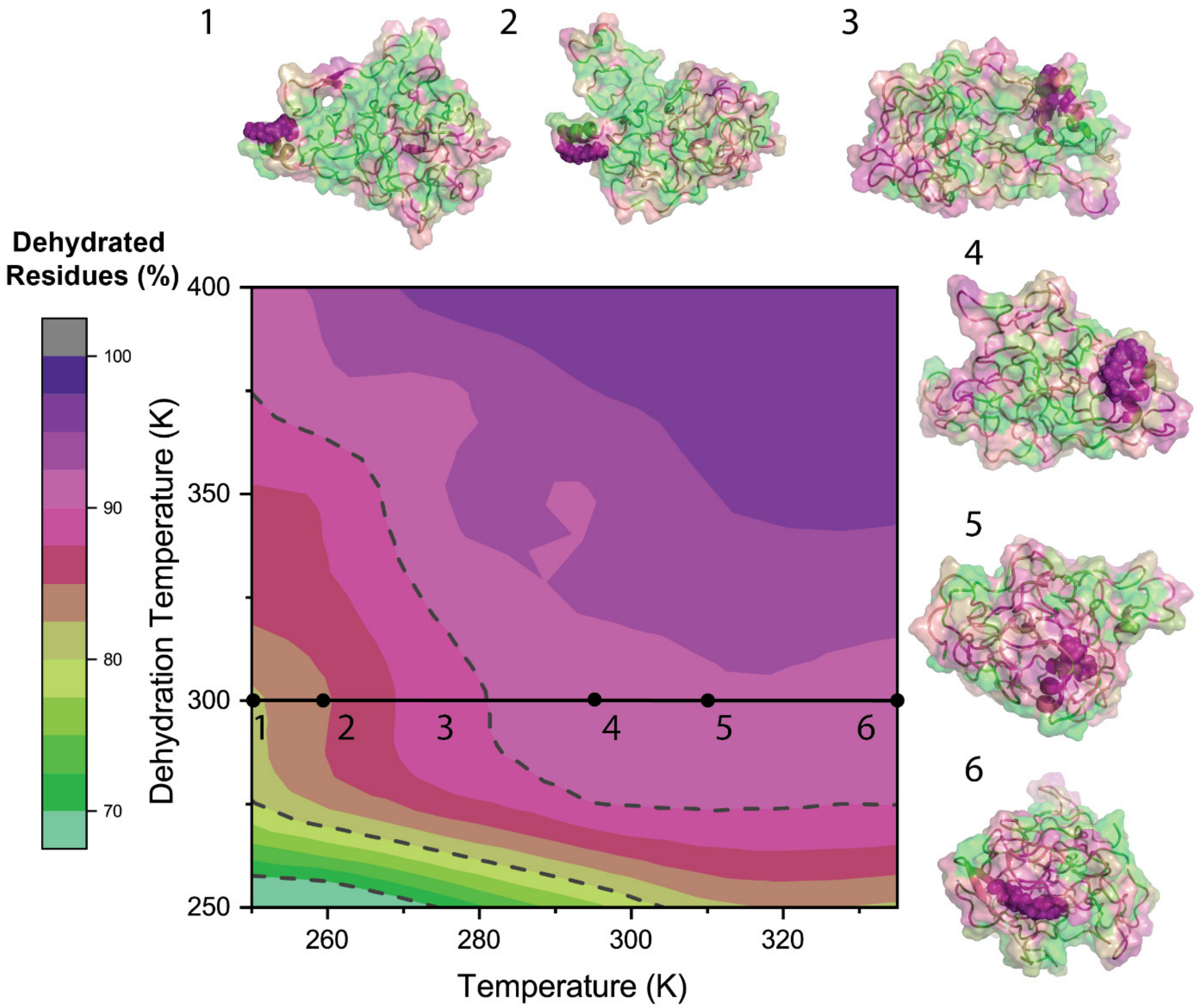

Figure S16. Lipidation induces temperature-dependent hydrophobic patches in myr-V_{60}. Contour plots represent the percent dehydrated residues for each FAME as a function of simulation (T_{MD}) and dewetting temperature (T_{dw}). The horizontal line is drawn to highlight the changes in structure and hydration of each construct as the temperature is increased. The protein surfaces are colored by residue according to the average number of water molecules in its hydration shell (purple = dehydrated, green = hydrated). Pentad repeats in the proximity of the lipid tails are more dehydrated compared to distal residues. Increasing the temperature alters the balance of interactions between lipid, protein, and water, leading to a structural rearrangement of the lipid tail.

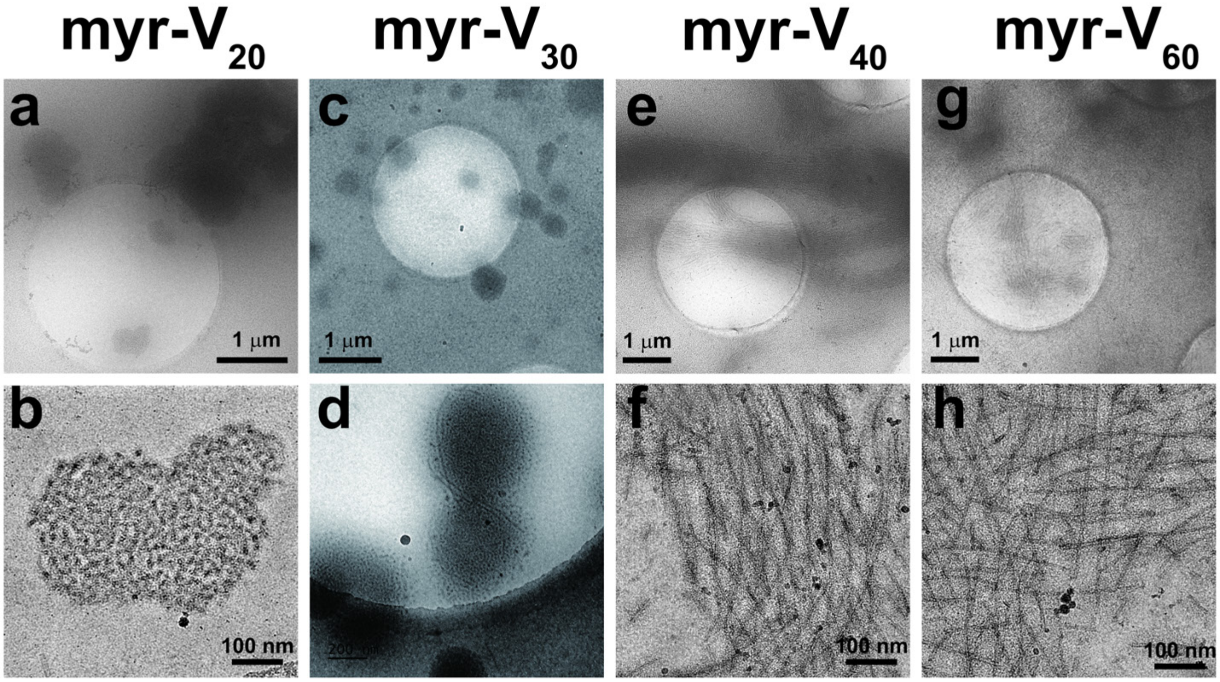

Figure S17. Nanoscale Characterization of FAME's coacervates internal morphology at 308 K using cryo-TEM. a,b) myr-V_{20}; **c,d)** myr-V_{30}; **e,f)** myr-V_{40}; and **g,h)** myr-V_{60}. At 308 K, DIC shows that FAMEs undergo liquid-liquid phase separation and form micron-sized coacervates (Figure 6). Cryo-TEM shows that these coacervates contain a large bundle of fibers with nanoscale diameters and large (micron size) lengths.

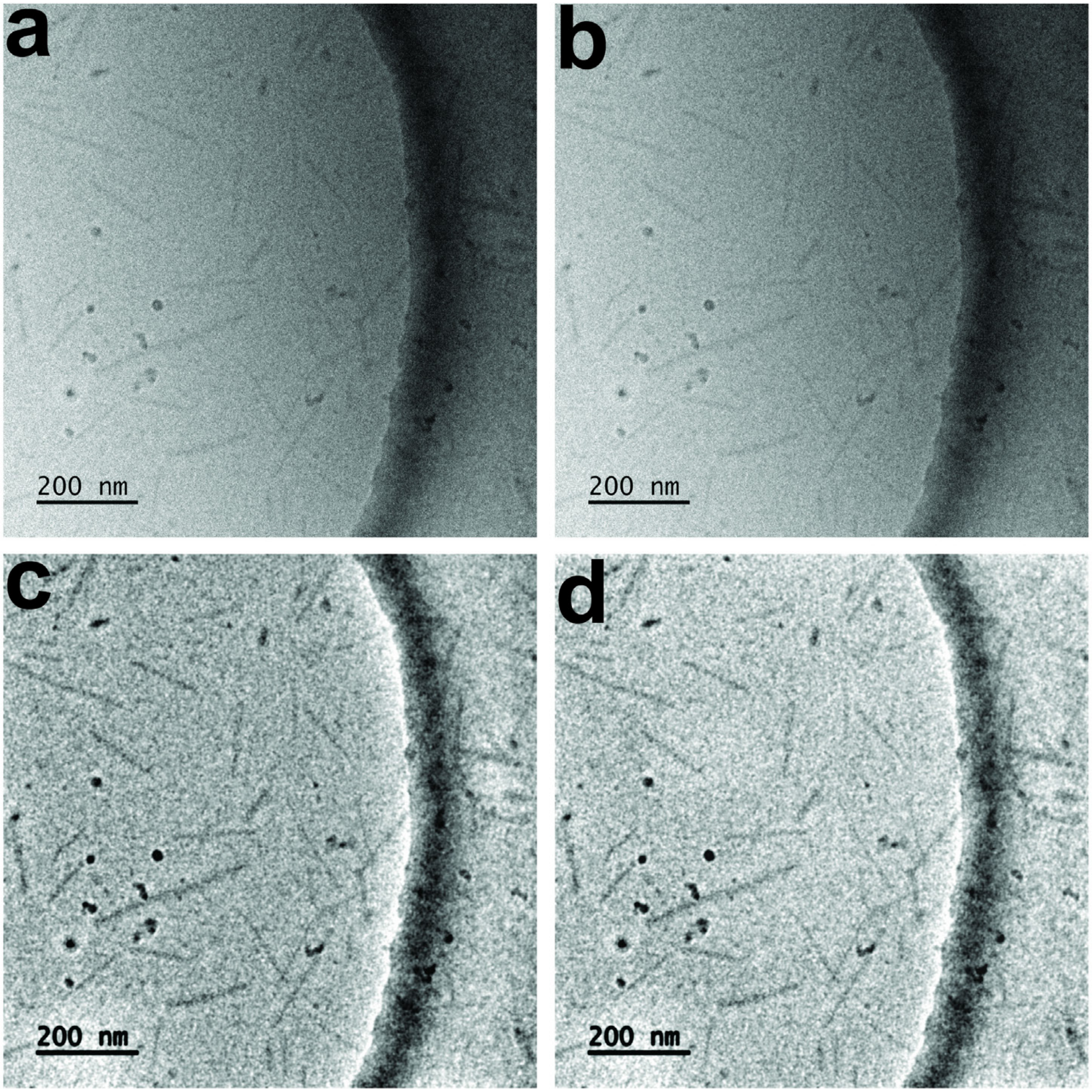

Figure S18. Raw and processed cryo-TEM micrographs of myr-V_{20} at 288 K (Figure 6a). a) Raw TEM micrograph; **b)** denoised with despeckle; **c)** processed with band-pass filter; **d)** after background correction.

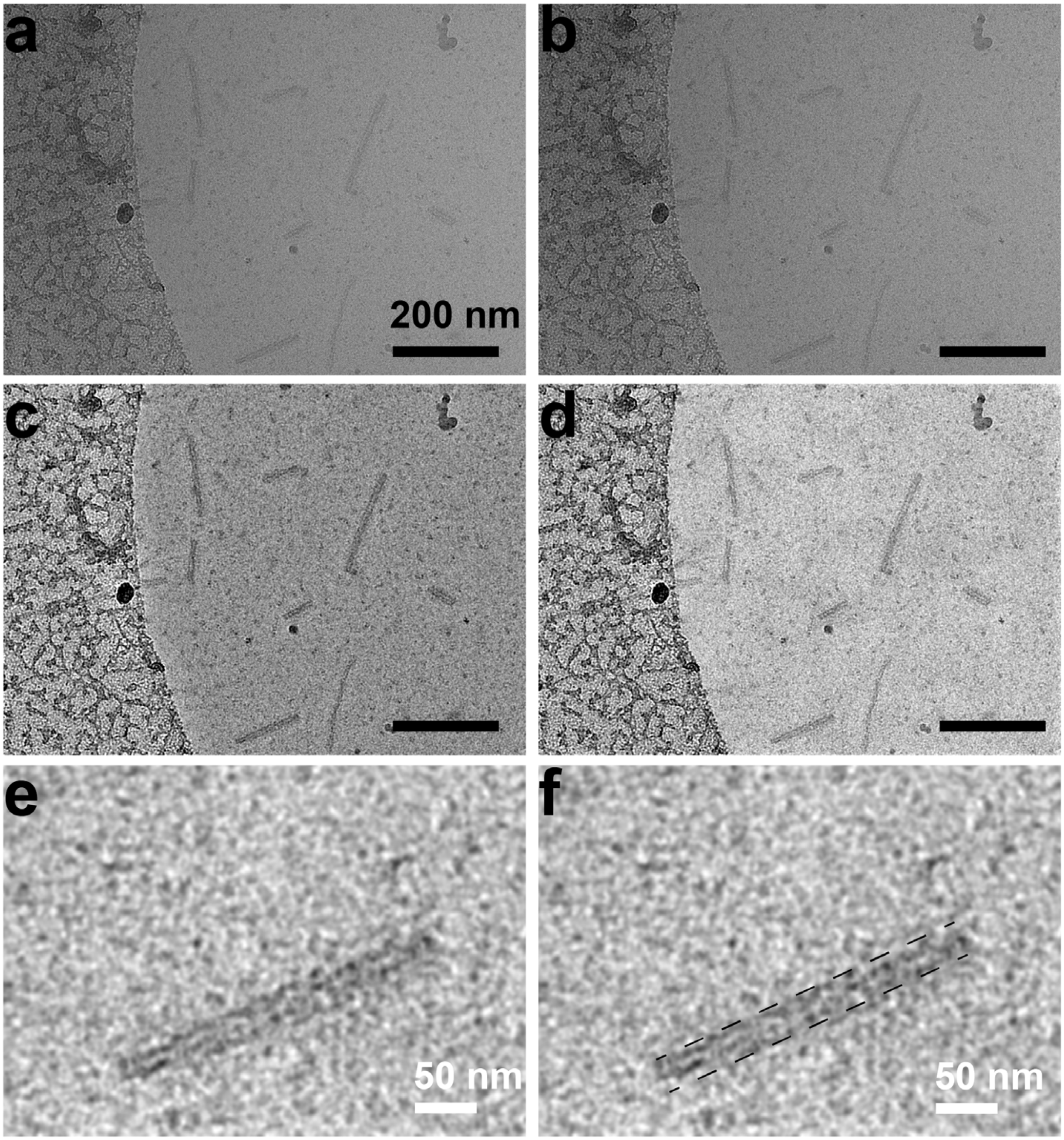

Figure S19. Raw and processed cryo-TEM micrographs of myr-V_{20} at 288 K after heating/cooling (Figure 6i). **a)** Raw TEM micrograph; **b)** denoised with despeckle; **c)** processed with band-pass filter; **d)** after background correction. **e,f)** Magnified view of a representative myr-V_{20} fibers. Dashed lines are added as visual guides for corona.

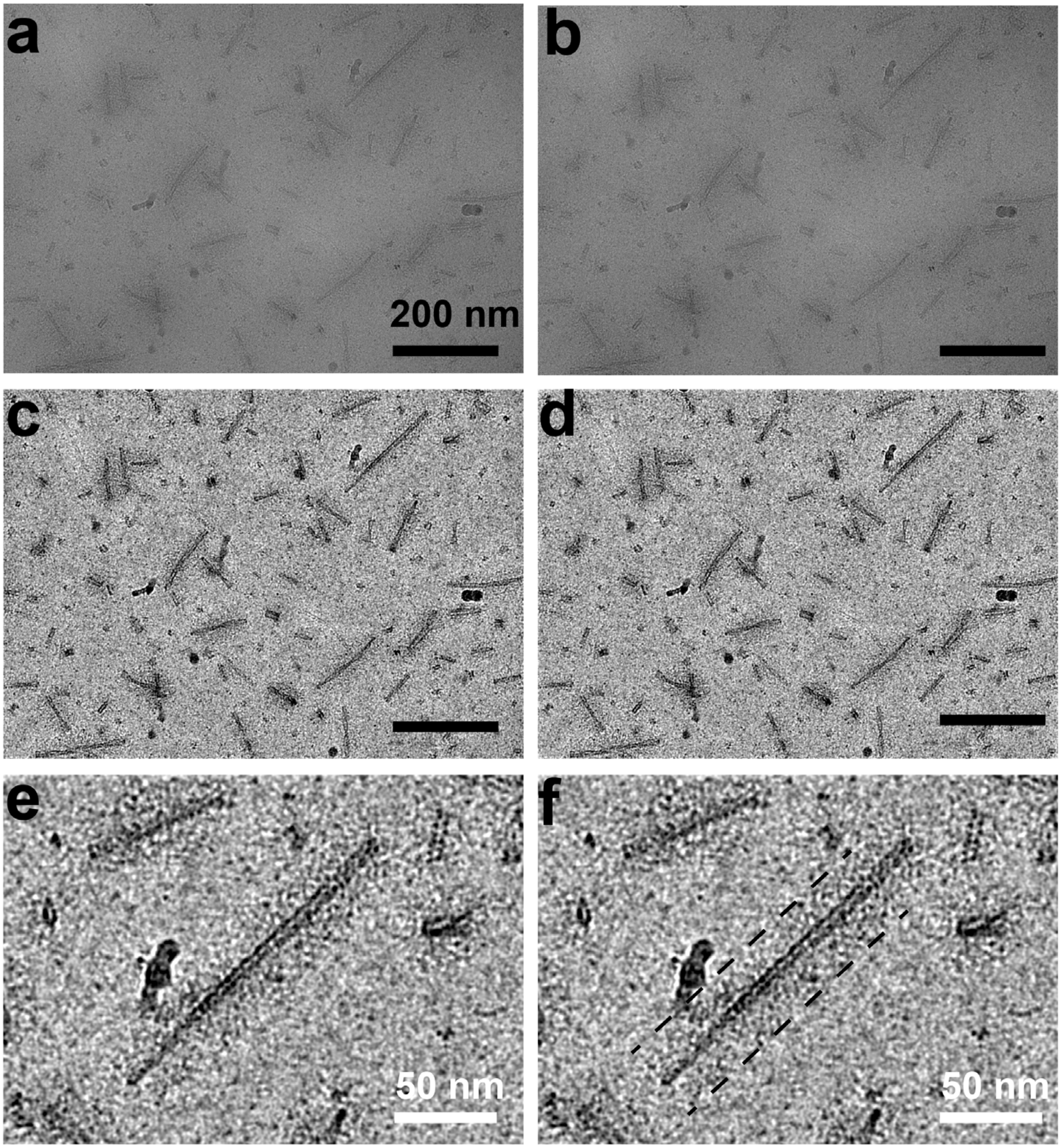

Figure S20. Raw and processed cryo-TEM micrographs of myr-V_{30} at 288 K after heating/cooling (Figure 6j). **a)** Raw TEM micrograph; **b)** denoised with despeckle; **c)** processed with band-pass filter; **d)** after background correction. **e,f)** Magnified view of a representative myr-V_{30} fibers. Dashed lines are added as visual guides for corona.

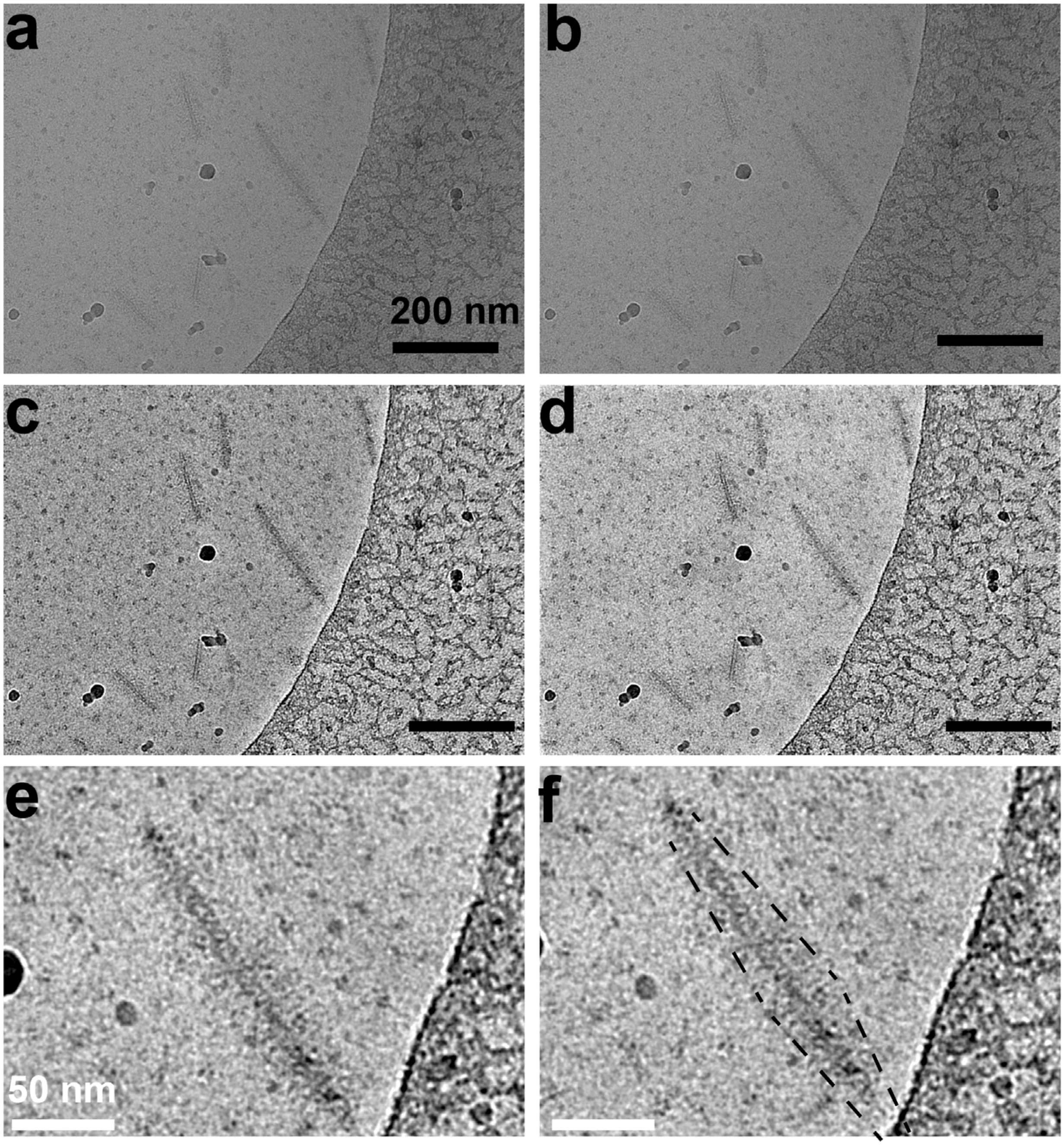

Figure S21. Raw and processed cryo-TEM micrographs of myr-V_{40} at 288 K after heating/cooling (Figure 6k). **a)** Raw TEM micrograph; **b)** denoised with despeckle; **c)** processed with band-pass filter; **d)** after background correction. **e,f)** Magnified view of a representative myr-V_{40} fibers. Dashed lines are added as visual guides for corona.

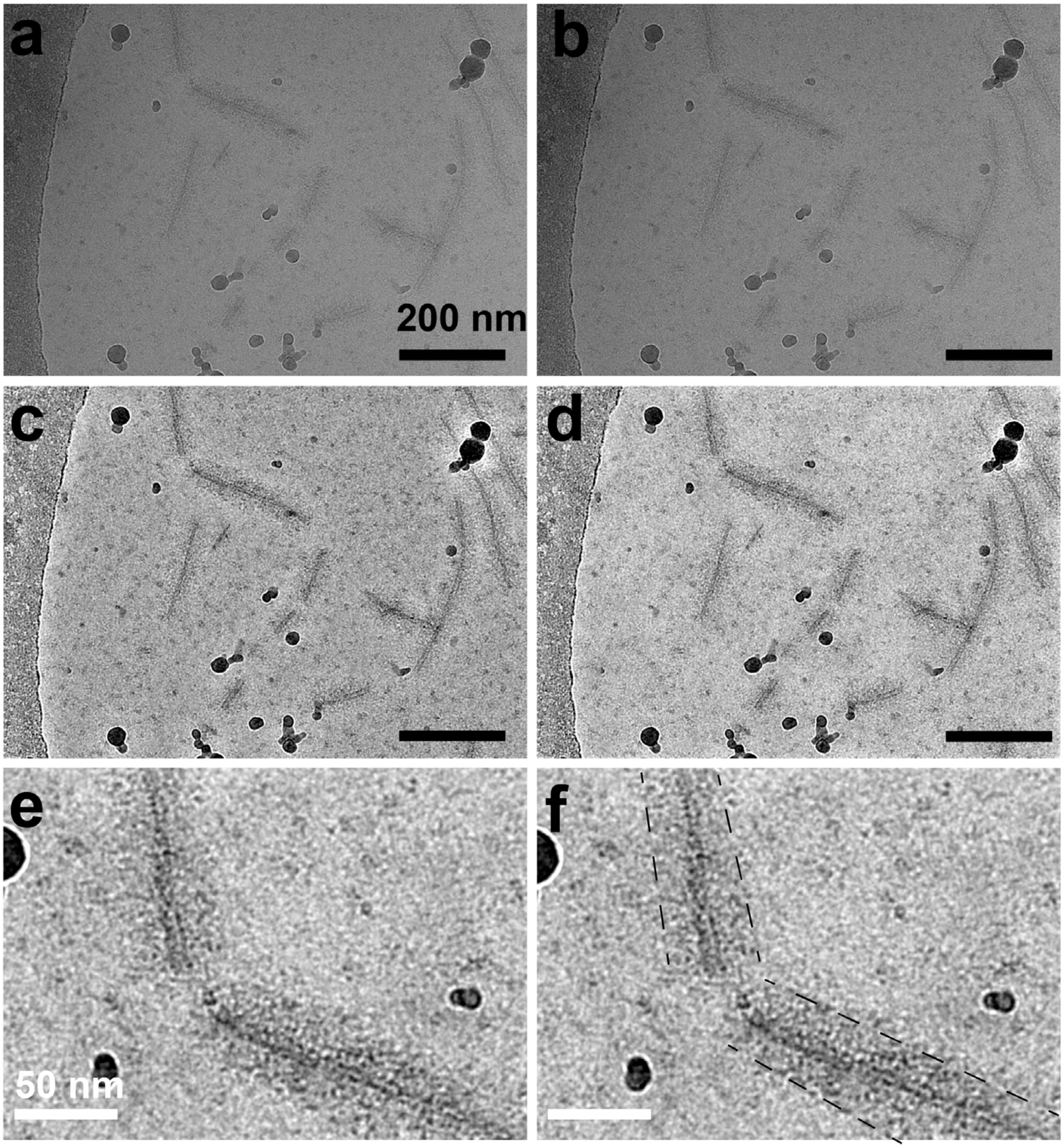

Figure S22. Raw and processed cryo-TEM micrographs of myr-V_{60} at 288 K after heating/cooling (Figure 6l). a) Raw TEM micrograph; **b)** denoised with despeckle; **c)** processed with band-pass filter; **d)** after background correction. **e,f)** Magnified view of a representative myr-V_{60} fibers. Dashed lines are added as visual guides for corona.

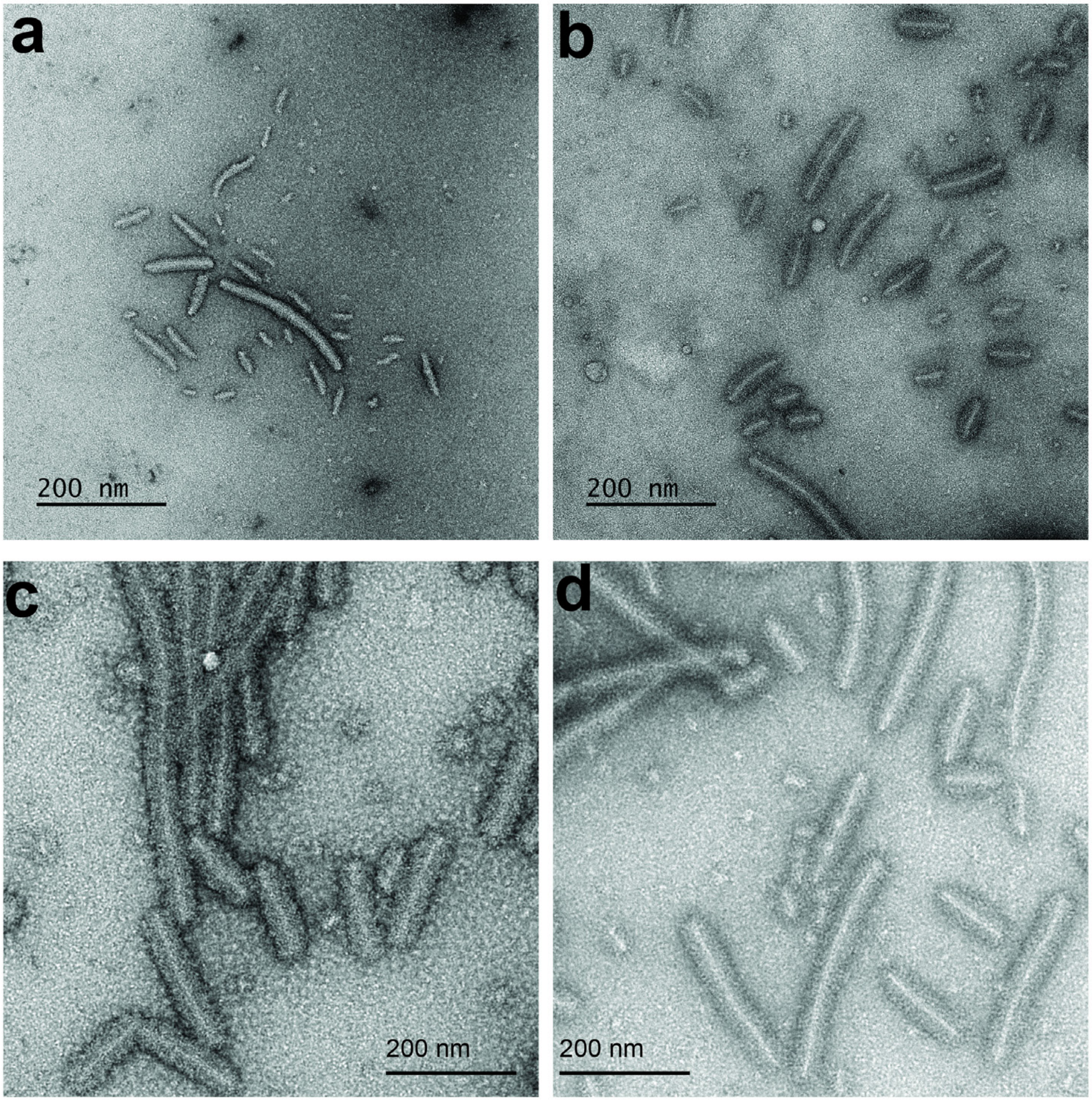

Figure S23. Negative-stained TEM is used to confirm the morphology and dimensions of FAMEs assembly after thermal treatment. a) myr-V_{20}; **b)** myr-V_{30}; **c)** myr-V_{40}; **d)** myr-V_{60}.

3. References

(1) Hossain, M. S.; Ji, J.; Lynch, C. J.; Guzman, M.; Nangia, S.; Mozhdehi, D. Adaptive Recombinant Nanoworms from Genetically Encodable Star Amphiphiles. *Biomacromolecules* **2022**, *23* (3), 863-876. DOI: 10.1021/acs.biomac.1c01314.

(2) Rudnick, D. A.; Johnson, R. L.; Gordon, J. I. Studies of the catalytic activities and substrate specificities of Saccharomyces cerevisiae myristoyl-coenzyme A:protein N-myristoyltransferase deletion mutants and human/yeast Nmt chimeras in Escherichia coli and S. cerevisiae. *J. Biol. Chem.* **1992**, *267* (33), 23852-23861.

(3) Žuvela, P.; Skoczylas, M.; Jay Liu, J.; Bączek, T.; Kaliszan, R.; Wong, M. W.; Buszewski, B. Column Characterization and Selection Systems in Reversed-Phase High-Performance Liquid Chromatography. *Chem. Rev.* **2019**, *119* (6), 3674-3729. DOI: 10.1021/acs.chemrev.8b00246 From NLM Medline.
